# Near-global freshwater-specific environmental variables for biodiversity analyses in 1 km resolution

**DOI:** 10.1038/sdata.2015.73

**Published:** 2015-12-08

**Authors:** Sami Domisch, Giuseppe Amatulli, Walter Jetz

**Affiliations:** 1 Department of Ecology and Evolutionary Biology, Yale University, 165 Prospect Street, New Haven, CT 06511, USA

**Keywords:** Freshwater ecology, Biogeography, Macroecology, Biodiversity

## Abstract

The lack of freshwater-specific environmental information at sufficiently fine spatial grain hampers broad-scale analyses in aquatic biology, biogeography, conservation, and ecology. Here we present a near-global, spatially continuous, and freshwater-specific set of environmental variables in a standardized 1 km grid. We delineate the sub-catchment for each grid cell along the HydroSHEDS river network and summarize the upstream climate, topography, land cover, surface geology and soil to each grid cell using various metrics (average, minimum, maximum, range, sum, inverse distance-weighted average and sum). All variables were subsequently averaged across single lakes and reservoirs of the Global lakes and Wetlands Database that are connected to the river network. Monthly climate variables were summarized into 19 long-term climatic variables following the ‘bioclim’ framework. This new set of variables provides a basis for spatial ecological and biodiversity analyses in freshwater ecosystems at near global extent, yet fine spatial grain. To facilitate the generation of freshwater variables for custom study areas and spatial grains, we provide the ‘*r.stream.watersheds*’ and ‘*r.stream.variables*’ add-ons for the GRASS GIS software.

## Background & Summary

Freshwater habitats cover only 0.1% of Earth’s surface, yet they provide habitat for ~10% of all animal species^1^. Many streams and lakes are considered biodiversity hotspots^2^, and understanding the biogeography of freshwater organisms is key for biodiversity conservation and management, especially in the context of ongoing and projected climate and land cover change^3^.

In addition to the macroevolutionary and biogeographic history of clades and past conditions, current-day species distributions are strongly determined by contemporary environmental factors. A variety of modelling tools, often summarized under the term of ‘environmental niche’ or ‘species distribution models’ have been developed to assess species ~environment relationships and to predict species geographic distributions. Here, the species geographic occurrences are related to the environmental conditions at those locations, yielding a model in environmental space that can then be projected and extrapolated into geographic space^4^. For successful modelling, in addition to reliable and ideally environmentally and geographically representative species occurrence records^5^, range-wide environmental data is needed. This usually comes in a spatially gridded format, where each grid cell is characterized by a continuous (e.g., temperature) or discrete (land cover class) variable. Over the past years a number of global high-resolution 1 km data sets have been created to assist the use of spatial modelling, such as gridded climate data from interpolated weather stations (WorldClim^6^), remote sensing products such as topography (SHuttle Elevation Derivatives at multiple Scales, SRTM^7^), or Moderate Resolution Imaging Spectroradiometer (MODIS) -derived products such as land cover^8^, and derivatives of those.

Appropriately quantifying species associations with their (abiotic) environment requires consideration of the full spatial extent of their occurrence^9^. While the inclusion of range-wide environmental data in models is becoming common place in the terrestrial and marine realm, it poses a real challenge in the freshwater realm^5^, since (i) the gridded site-level (terrestrial) environmental variables do not translate well into the directional freshwater ecosystems without accounting for the down-stream connectivity, and (ii) even if freshwater-specific environmental data are available, they are mostly restricted to single basins and watersheds, or political borders^5,10^. Likewise, the lack of range-wide freshwater-specific environmental information hampers comparable freshwater biodiversity and ecosystem analyses in general, such as metapopulation and -community models, ecosystem or ecoregion delineations, and functional biodiversity assessments^11^.

To facilitate a more geographically inclusive and comparable studies in freshwater biodiversity science, we developed near-global 1 km gridded freshwater-specific information comprised of multiple, complementary environmental variables of known relevance for species distributions: climatic (monthly air temperature and precipitation), topographic (elevation and slope), land cover, surface lithological and soil variables (Data Citation 1). This newly developed information is based on the 1 km HydroSHEDS hydrography^12^ (**Hydro**logical data and maps based on **SH**uttle **E**levation **D**erivatives at multiple **S**cales, www.hydrosheds.org) and accounts for the upstream connectivity within the stream network. Each environmental variable is computed for each single 1 km stream gird cell individually along the stream network^10^, by (i) delineating the upstream sub-catchment for each grid cell, and (ii) summarizing and relating the upstream environmental conditions of each variable to each stream grid cell along various metrics (upstream minimum, maximum, average, sum, weighted average, sum and weighted sum, Table 1). This procedure integrates the connectivity, and consequently the upstream environmental conditions can be traced along the stream network (for instance, percent upstream forest cover). In addition, (iii) we extended the data to lakes and reservoirs of the 1 km gridded Global Lake and Wetlands Database^13^ and unified the stream and lake data layers for each variable.

The newly developed layers facilitate—for the first time—large-scale models of the distribution and community characteristics of freshwater biota. While the layers (Data Citation 1) can be used ‘as is’, we also developed the GRASS-GIS^14^ add-ons ‘*r.stream.watersheds*’ and ‘*r.stream.variables*’ that allow the re-calculation of specific environmental variables for a given study area or different spatial grain in an automated and parallelized manner. A subset of the layers can also be visualized online at www.earthenv.org/streams (Data Citation 1).

## Methods

### Base layer preparation

We used the HydroSHEDS 30 arc sec (hereafter referred to as 1 km) hydrography^12^ as a basis for all computations. HydroSHEDS is based on the SRTM^7^ Digital Elevation Model (DEM), and consists of a gridded drainage direction layer at 15 arc sec spatial grain (~500 m) and a vectorised near-global stream network (with a minimum of 100 upstream cells), and the upscaled products on a 1 km spatial grain^15^. The advantage of using the 1 km stream network for our analyses (as opposed to 500 m) was that it allowed aligning its spatial grain with the environmental source layers to create the freshwater-specific variables, avoiding uncertainties resulting from downscaling data at multiple resolutions^16^.

As the stream network was originally computed in ESRI ArcGIS^17^, we started the computation using this software and merged single continents regarding the vectorised stream network and the void-filled Digital Elevation Model (DEM) to single near-global layers (note that the SRTM^7^ and hence the HydroSHEDS data does currently not exceed 60°N northern latitude^12^). Here, the use of the ESRI ArcGIS software ensured the alignment of the DEM and streams, avoiding pixel mismatching and geographic projection issues. The stream network was then transformed from 1 km vector data to 1 km grids to facilitate the subsequent computation, while being inclusive as no ‘corner cutting’ was performed (where diagonal cells could have an additional adjacent cell due to possible inaccuracies in the 1 km DEM^12^). We then used the flow direction layer from HydroSHEDS and identified the Strahler stream order^18^ of each stream grid cell (Spatial analyst toolbox, Hydrology, Stream Order^17^). The DEM and stream order layer for the entire stream network were then imported into GRASS GIS 7.0 (ref. 19) under the Linux environment for all subsequent processing steps.

To ensure that the water is flowing downstream *in* the stream channels, we carved the stream network into the DEM by a depth of 22 m. This carving depth was chosen after a step-wise increase of the carving depth by 2 m and by checking for the downstream connectivity along the water courses (note that this carved elevation layer was not used for any elevation-based analyses later on). Running a 3×3 cell moving window analysis (*r.neighbors* function in GRASS^14^) along each stream order separately allowed us to remove coarse sinks and peaks, while possible remaining minor pits and peaks were smoothed using the *r.hydrodem*-function. We then calculated a new flow direction layer based on the carved DEM using the *r.watershed*-function, enabling water to flow into multiple down-stream cells (−MFD flag), forcing a positive flow accumulation for potential underestimates (−a flag) and emphasizing the flow accumulation in flat areas (−b flag). This layer needed to be created to avoid any incompatibilities between flow direction layers deriving from GRASS and the one originally created in ArcGIS.

All subsequent calculations were processed in parallel using the High Performance Computing facility at Yale University in compressed chunks of 10,000 files. This was done to maintain a fast I/O load and to avoid the overload of index nodes filling up the hard disk due to the high number of small files. To optimize the parallelization, we split the global stream network again into six continents (North and Central America, South America, Europe, Africa, Asia and Australasia, following the original divisions of^12^), and extracted the coordinates of the gridded stream network along a unique grid ID (hereafter ‘gridID’ layer). Each single continent and the IDs served as a template for creating the final layers later. In total, the entire near-global stream network consisted of 20,794,251 grid cells, and the spatial extent for all analyses ranged from 60°N to 5°S latitude, and 145°W to 180°E longitude (i.e., the spatial extent of HydroSHEDS).

### Sub-catchment delineation

The flow direction layer served as a basis for delineating the upstream sub-catchment of each 1 km stream grid cell using the *r.water.outlet* -function in GRASS^14^, where each stream grid cell along the stream network served as a pour point (see Fig. 2a). This is considered the most intense computation process because each sub-catchment needs to be delineated individually based on the flow direction layer, i.e., each outlet needs to ‘find’ its catchment across the near-global flow direction layer (we used the global layer here to avoid any truncation of sub-catchments located between continents). Besides storing the sub-catchment for each grid cell as a gridded GeoTIFF layer, we also extracted the stream network (water courses) within each sub-catchment separately. For each of these two files—sub-catchment and upstream water courses—we calculated the distance from the outlet to each grid cell within the specific sub-catchment with the grid cells as spatial units (‘as the crow flies’ and ‘as the fish swims’, respectively). These distances were used to compute an inverse distance weighting factor (weighting factor=1/distance) to create distance-weighted averages of the variables (see below). Thus, the environment in and in the immediate vicinity of a given outlet grid cell is considered to have the most influence on the focal grid cell, and this influence decreases with an increasing distance from the given grid cell (Fig. 2a). These three layers (sub-catchment, and the two distance layers) served as a basis for the calculation of the stream variables for each grid cell.

### Source layers of the stream variables

To create the stream variables we used globally available and spatially continuous gridded environmental data at a native spatial grain of 1 km as source layers (Table 1). These were (1) interpolated climate from the WorldClim^6^ data set (monthly minimum and maximum air temperature, and the monthly sum of precipitation), (2) topography based on the HydroSHEDS void-filled DEM^12^ (elevation, slope, flow accumulation and flow length as the sum of contributing grid cells), (3) land cover derived from the consensus land-cover product^8^ (representing the percent cover for each of the 12 classes in a given grid cell), (4) surface geology^20^ (where each of the 92 discrete classes represents the approximate geological age), and (5) 10 soil classes from the SoilGrids1km data base through ISRIC/WDC-Soils^21^.

Because the spatial extent of the climate layers at the coasts were slightly smaller than the spatial extent of the HydroSHEDS stream network due to the upscaling procedure^15^, we extended the climate layers by 15 grid cells into the oceans (*r.grow* function) to cover all stream cells and to avoid gaps and truncated coastal streams in the original stream network. The originally discrete surface geology layer was transformed into 92 binary 0–1 variables to facilitate the computation. Predictions regarding the original soil types in the SoilGrids1km database have been produced only for areas with vegetation cover and urban areas. No estimate is provided for shifting sands/deserts and permanent ice areas^21^. For the sake of consistency regarding the extent and number of grid cells (and NoData cells) we replaced the missing values in these areas with zero.

### Derived metrics from the source layers

For each sub-catchment, we first overlaid each source layer and clipped all areas not covered by the sub-catchment. We then calculated various metrics of the remaining portion of each source layer that covered the sub-catchment (Table 1), including the minimum, maximum, average, sum, and distance-weighted average and distance-weighted sum of the upstream values using the *r.univar* function and custom code. All units were kept from the source layers (note that temperature values and slope values are multiplied by 10 and 100, respectively, to keep integers and to reduce the file size, see Table 1 and Table 2 (available online only).

**Figure 1:**
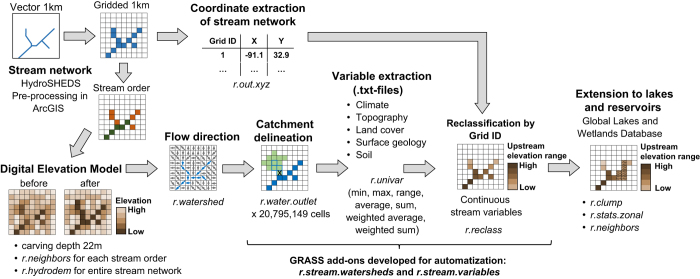
Schematic overview of the main steps for creating the freshwater variables and the *GRASS GIS*^14^ *functions* used. First the vectorised HydroSHEDS^12^ stream network was transformed into grids and the stream order computed, and this layer was used to recondition the Digital Elevation Model^12^ (DEM). The corrected DEM was then used to calculate the flow direction and to delineate the upstream sub-catchment for each cell along the stream network. Various metrics (min, max, range, average, sum, weighted average and weighted sum) were extracted as text-files from existing climate^6^, topography^12^, land cover^8^, surface geology^20^, and soil^21^ data sets, where each text-file contained the different metrics of a given variable. Once all catchments were processed, the text-files were merged and reclassified into a spatial grid, representing continuous upstream variables. Finally, lakes and reservoirs were extracted from the 1 km gridded Global Lakes and Wetlands Database^13^ and the variables were averaged across each single lake and reservoir entity that intersected with the stream network.

All derived metrics were stored for each cell along the stream network, and then merged into near-global GeoTIFF layers using the gridID layer (see Fig. 1). Finally, the upstream averaged and weighted averaged climatic layers were further processed into 19 long-term ‘hydro-climatic’ variables following the bioclim framework^22^ using monthly temperature and precipitation.

### Extension to lakes and reservoirs

**Figure 2:**
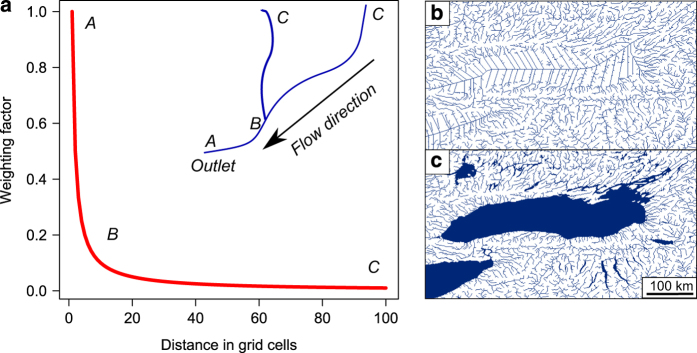
(**a**–**c**) Inverse-distance weighting and integration of lakes and reservoirs. (**a**) Scheme for obtaining the inverse distance weighting factor for calculating the weighted average and sum of the variables. The position of the letters in the stream network (inset) correspond to their approximate position on the curve. (**b**,**c**) Illustration of the modified HydroSHEDS^12^ stream network before (**b**) and after (**c**) integrating the lakes and reservoirs of the Global Lakes and Reservoirs Database^13^.

The HydroSHEDS data set does not contain lakes and reservoirs *per se*, although the Global Lake and Wetlands Database^13^ (GLWD, 1 km spatial grain) was integrated during the computation of the hydrography^12^. In other words, the GLWD data set has been used to create the hydrography^12^ in terms of the geographic location (thus the lakes and reservoirs spatially match with the stream network), however they are not marked as such in the stream network. The lakes and reservoirs can be identified by a ‘fish-bone’ structure (Fig. 2b,c) due to zero slope where the flow accumulation algorithm is forced to work properly.

We extracted the lakes and reservoirs with a surface area >0.1 km^2^ from GLWD to extend the newly developed stream variables also to lentic areas along the river network. We first identified all lakes and reservoirs that represent single spatial units (*r.clump*—function), and then overlaid and averaged the newly developed variables (Data Citation 1) over each unit to mimic the more static environmental condition in standing waters (*r.stats.zonal* -function). Possibly steep drops at the interface of stream and lake/reservoir grid cells were smoothed by averaging the values in these grid cells at the interface within a 3×3 cell neighbourhood at these locations.

**Figure 3:**
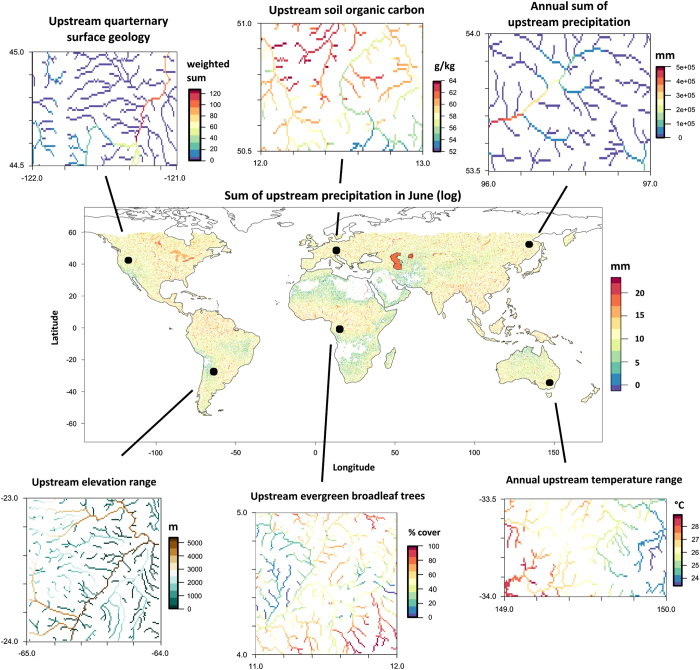
Example maps of the newly developed freshwater variables (Data Citation 1). A global overview of the natural log-transformed sum of precipitation in June (grid cells are aggregated by factor 4 for a better visualisation), and insets representing climate, topography, land cover, surface geology and soil (at the original units and 1 km spatial grain).

In summary, the entire procedure resulted in the computation of stream-specific variables with a continuous surface along the river continuum^23^ (Fig. 3), and the variables were subsequently extended to lakes and reservoirs by averaging the upstream environmental conditions for each lake/reservoir connected to the stream network.

### Code availability and GRASS GIS add-ons

We developed the ‘*r.stream.watersheds*’ and ‘*r.stream.variables*’ add-ons for GRASS GIS^19^ for users who wish to calculate additional variables or metrics, and to apply the method for a specific study area on a different spatial grain. The add-ons automatize and parallelize the main processing chain with a user-specified number of cores, and can be downloaded from the GRASS repository (http://grass.osgeo.org/download/addons/). See the Supporting Information for exemplary code and the tutorial on spatial-ecology.net.

The ‘*r.stream.watersheds*’ add-on delineates the sub-catchment for each stream grid cell and saves the single sub-watershed, and the stream sections within each sub-watershed as GeoTIFF raster files in zipped folders on the hard disk. Users need to provide (i) a flow direction layer generated in GRASS GIS (*r.watershed*) and (ii) a gridded stream network layer. Once this add-on has finished, the second ‘*r.stream.variables*’ add-on can be used: it takes the output from ‘*r.stream.watersheds*’ and overlays the single sub-watersheds with environmental variables to calculate various user-specified metrics simultaneously (number of contributing cells, minimum, maximum, range, average, sum, standard deviation, coefficient of variation). The final output for each metric is a contiguous GeoTIFF layer that is ready to use in a GIS software or e.g. species distribution modeling application. Note that all output layers are stored as integers (Int32 datatype) to reduce the file size, and users can set a scale factor to avoid the truncation of decimals.

The add-ons take advantage of multiple processors to speed up the calculation. For instance, using 8 processors on a 2.66 GHz PC for a stream network that consists of ~90,000 grid cells takes ~1.5 h for the watershed delineation, and ~30 min for each input layer (yielding up to eight output layers).

## Data Records

All newly developed 1 km variables are available as compressed netCDF-4 layers on a near-global extent at www.earthenv.org/streams (Data Citation 1) and the Dryad Digital Repository (Data Citation 2). In addition, a visualisation of the layers is given online at www.earthenv.org/streams (Data Citation 1), where users can browse a subset of variables in each category at the stream level and in 1 km resolution.

We provide a variety of different metrics of the upstream environment along the stream network, resulting in a total of 324 layers (Table 1 and Table 2 (available online only)).

Each variable comes in the netCDF-4 format (network Common Data Form version 4) in a cell size of 0.0083333333° (30 arc-seconds, i.e., 30/3,600 of a degree) in the WGS84 coordinate system with an extent of 60°N to 5°S latitude and 145°W to 180°E longitude. All variables consists of 39,000 columns and 13,920 rows. To reduce the file size for download, each netCDF file contains one variable (e.g., upstream average land cover) where the single layers (e.g., 12 landcover classes) are stacked as single bands using the software NCO (ref. 24). The pixel type of the layers ranges from Byte (upstream land cover) to Float64 (upstream precipitation).

All variables were computed based on i) average or sum, or ii) inverse distance-weighted average or distance-weighted sum over the upstream network (except for surface geology where we only computed the distance-weighted sum under the assumption that the impact is dampened along the river continuum, opposed to the continuous accumulation along the river continuum). Regarding the topography, land cover and soil, we also computed the minimum, maximum and the range of the upstream values. Please see Table 2 (available online only) for a full description of all available layers.

**Climatic variables** consist of (i) 12 monthly minimum and maximum temperature and precipitation variables, and (ii) 19 long-term hydroclimatic variables following the bioclim framework^22^. For each, we computed the average and weighted average for temperature variables (units in °C * 10), and the sum and inverse distance weighted sum for precipitation variables (mm). Likewise, the hydroclimatic variables consist of the monthly average and sum, and the monthly inverse distance weighted average and sum for the aggregations (e.g., upstream sum of precipitation during the warmest quarter). The climate data derives from the WorldClim data base^6^, consisting of monthly gridded climate data interpolated between point locations across the globe, and averaged across the years 1950–2000 on a 30-arc-second spatial grain.

The **stream topographic variables** consist of elevation (m), slope (° * 100), flow length and flow accumulation. Elevation and slope data were aggregated using the upstream minimum, maximum, range and average aggregation techniques. Flow accumulation and flow length are computed as the sum of contributing (1 km) grid cells for the entire sub-catchment and only regarding the stream network within the sub-catchment, respectively. The source data for these variables derives from HydoSHEDS^12^ (30-arc-second spatial grain) which in turn is based on SRTM^7^ data from the year 2000.

**Landcover variables** consist of 12 classes and depict the upstream percent coverage of a given landcover class: Evergreen/Deciduous Needleleaf Trees, Evergreen Broadleaf Trees, Deciduous Broadleaf Trees, Mixed/Other Trees, Shrubs, Herbaceous Vegetation, Cultivated and Managed Vegetation, Regularly Flooded Vegetation, Urban/Built-up, Snow/Ice, Barren, Open Water). For each class we computed the upstream minimum, maximum, range, average and inverse distance weighted average percent cover. The source data for these variables is the Consensus Landcover dataset^8^ with a temporal coverage of 14 years (1992–2006) on a 30-arc-second spatial grain.

**Surface geological** variables consist of 92 variables indicating the geological age of a given sub-catchment based on the surface geology. Here, the data aggregation type used was the inverse distance-weighted sum of grid cells of a given surface geology type. The source data is derived from USGS^20^ via the worldgrids.org portal on a 30-arc-second spatial grain, and the data acquisition period of the source data covers the timeframe from 1960–2000.

The **soil variables** consist of the upstream minimum, maximum, range, average, and inverse distance-weighted average of ten soil variables that were predicted within the standard depth of 2.5 cm (0–5 cm standard thickness): soil organic carbon (g/kg), soil pH (pH * 10), sand content mass fraction (%), silt content mass fraction (%), clay content mass fraction (%), coarse fragments (>2 mm fraction) volumetric (%), cation exchange capacity (cmol/kg), bulk density of the fine earth fraction (kg/m^3^), depth to bedrock (R horizon) up to maximum 240 cm (cm), predicted probability of occurence (0–100%) of R horizon across sub-catchment. These variables derive originally from the soilgrid.org database (ISRIC)^21^, where the spatially contiguous soil types are derived from predictions. The raw data for the predictions consist of observations at point locations from a 55 year time period (1950–2005). Note that the predictions on a 30-arc-second spatial grain have been produced only for areas with vegetation cover and urban areas, and no estimates are provided for shifting sands/deserts and permanent ice areas^21^ (see http://www.isric.org/content/technical-specifications-soilgrids). For the sake of consistency regarding the extent and number of grid cells (and NoData cells) we replaced the missing values in these areas with zero.

## Technical Validation

### Quality control of the sub-catchment delineation

We checked for errors in the variables (Data Citation 1) that may arise from incorrect downstream routing of the flow accumulation prior to the extension to lakes and reservoirs. Possible reasons for an incorrect routing could be (i) remaining pits and peaks in the DEM (note that a more extensive ‘cleaning’ of the DEM based on a fixed threshold in one area can lead to an incorrect flattening in other areas); (ii) possible inaccuracies and intermittences in the underlying stream network^12,15^ due to the 1 km spatial grain (since the HydroSHEDS stream network was upscaled from the original 90 m spatial grain), (iii) cells that are located at the 60°N latitude boundary may have a truncated catchment, (iv) coastal areas that have a very flat/constant elevation without any gradients. Moreover, (v) while the stream network layer was kept ‘inclusive’, i.e., all stream cells from the HydroSHEDS river network were kept for our analyses, this also required addressing to deal with these cells where the water flow could have been bypassed during the catchment delineation (no watershed was created). In addition, (vi) the differences in flow direction algorithms between GRASS and ArcGIS to compute the upstream watersheds can differ, leading to slightly different flow direction maps.

These cells were identified for each stream order separately through their lower than expected upstream flow accumulation given the stream order. For instance, a sub-catchment of a 2nd stream order needs to consist of at least three cells (two 1st order cells merging to a 2nd order cell). This scheme was applied to all stream orders, where the minimum number of upstream grid cells follows the hierarchy of Strahler’s stream order^18^ (with a minimum required number of 3, 7, 15, 31, 63, 127, 255, and 511 grid cells for 2nd to 9th order streams, respectively).

In total 314,084 grid cells were identified to have a truncated sub-catchment, which corresponds to 1.5% of all stream grid cells on the near-global extent (the layer ‘missing_cells.nc’ is provided in the ‘quality_control.nc’ file, Table 2 (available online only)). The values for each environmental variable in these cells were corrected by first assigning the maximum value of the surrounding 3×3 cell neighbourhood matrix for each stream order separately. This reduced the number of cells with incorrect values to 6,924, i.e., 97.8% of initially incorrect cells could be filled. However, as not all cells could be corrected, this also meant that their immediate neighbourhood was assigned incorrectly, and that the flow direction was routed incorrectly (note that these cells occurred mainly in flat areas such as in lakes or coastal areas). A second round of correction was undertaken by repeating this procedure iteratively in the neighbouring cells until all remaining incorrectly assigned cells were filled.

While this procedure forced calculated layer values (e.g., upstream average temperature) into stream reaches that would be otherwise underestimated, it left a counterpart of stream cells with overestimated values. To identify these river reaches, we overlaid the flow accumulation and stream order layers in Google Earth Engine with the global satellite topography imagery (TruEarth 15 m), and visually checked whether the upstream flow accumulation matches with the network topology in the satellite image and the stream order. In total, 3301 manual corrections were made globally, all near coasts, since these were the areas where the flow accumulation routing could fail due to flat areas. We decided to omit these stream cells from all layers due to high uncertainties in the flow direction, and provide a separate layer to users to identify these cells (‘cells_removed.nc’ in the ‘quality_control.nc’ file, Table 2 (available online only)).

### Validation

#### Background

Among the most important predictors for delineating freshwater species distributions are water temperature and discharge^5^.

Developing continuous stream layers directly addressing these variables would be ideal, but requires spatially and especially temporally continuous and detailed, high-quality information such infiltration rates, evapotranspiration rates, snow/ice cover and melt, and direct anthropogenic water abstraction and water use. These data are however only partially available on the required spatial and temporal resolution. Thus, our aim here is to validate the overall pattern of the variables we were able to produce, and to show how upstream processes influence those located more downstream over large spatial scales but fine grains via the transport characteristics of the stream network.

**Figure 4:**
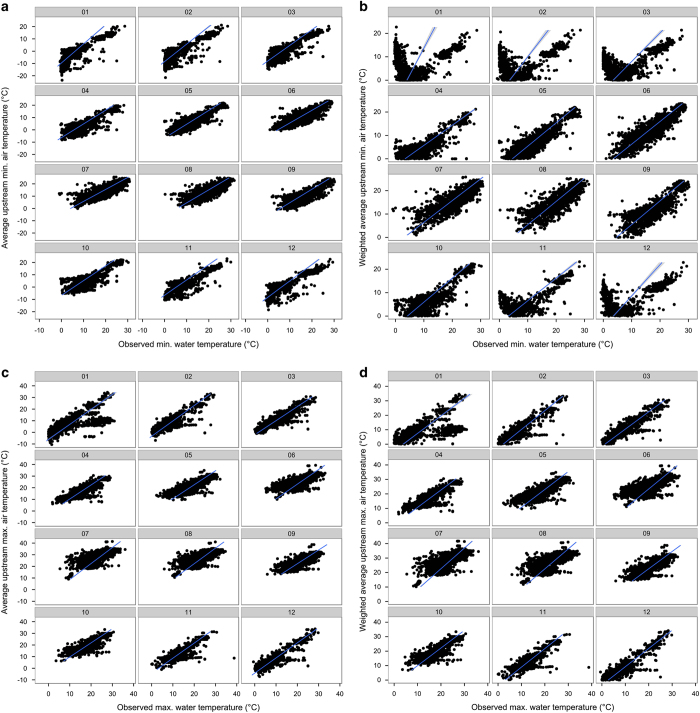
(**a**–**d**) Relationship between observed stream temperature and newly-developed temperature variables. Observed monthly minimum (**a**,**b**) and maximum (**c**,**d**) stream temperature plotted against the upstream average (**a**,**c**) and distance-weighted average (**b**,**d**) air-temperature for each month (plot headers correspond to January to December). Lines correspond to the fit of the linear regression with 95% confidence intervals.

**Figure 5:**
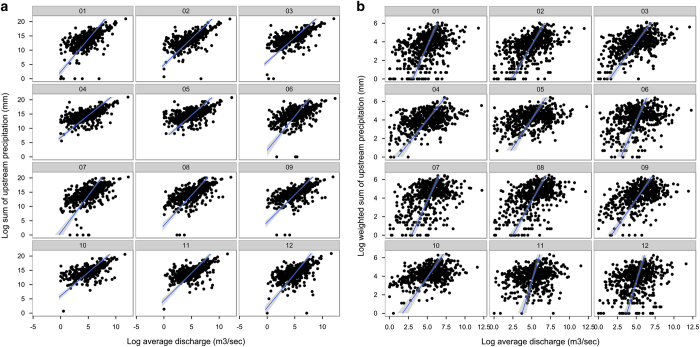
(**a**,**b**) Relationship between observed discharge and newly-developed precipitation variables. Natural log-transformed observed monthly average discharge plotted against the natural log-transformed sum (**a**) and distance-weighted sum (**b**) of upstream precipitation for each month (plot headers correspond to January to December). Lines correspond to the fit of the linear regression with 95% confidence intervals.

We therefore assessed how well the newly-developed monthly upstream temperature and precipitation layers may be suitable proxies for these variables. Specifically, we assessed how well local measurements of monthly water temperature and discharge^25–27^ (Supplementary Fig. S1a,b) are predicted by the newly-developed freshwater variables (Data Citation 1) and how much this fit was improved when using upstream *average* or *distance-weighted average* variables instead. We plotted the relationships between observed data and the variables and used the R^2^ from linear regressions (in log space) as a measure of goodness of fit (Table 3 and Figs 4 and 5, precipitation and discharge data were log-transformed for plotting). All calculations were done in the software R (ref. 28).

#### Monthly upstream temperature versus observed monthly water temperature

The observed stream temperature data was compiled from various sources (National Water Quality Monitoring Council^25^, the GloRICH data base^26^ and from the Environment Agency^27^, Supplementary Fig. S1a) and aggregated to monthly minimum and maximum values to match the temporal resolution of the temperature variables (Data Citation 1). First, only observations from 1950–2000 were used as this matches the timeframe of the WorldClim^6^ data set. Second, at least three observations per month for three years were required, resulting in a minimum of nine observations that were averaged for each site. This yielded 319 unique locations with 1740 observations that were moved to the closest grid cells in the stream network using a ‘snapping’ distance of 3 km using ‘RasterTools’ ^29^. This distance was chosen due to possible spatial inaccuracies in the stream network while retaining as much of the data as possible for the validation (Supplementary Fig. S1a). We then extracted the upstream average and distance-weighted average of the temperature variables at those locations.

Goodness of fit (R^2^) regarding the minimum monthly temperature was higher from November to March (ranging from 0.57 to 0.72) when the upstream average temperature was used (as opposed to the distance-weighted temperature), and the opposite pattern was found from May until October (R^2^ ranges from 0.64 to 0.75, Table 3, Fig. 4).

For maximum monthly temperature the pattern was reversed, and the distance-weighted averaged values had in general a higher goodness of fit during the winter months (R^2^ ranged 0.44–0.84) than the evenly averaged air temperature (Table 3). During the summer months, the averaged values indicated a better fit than the distance-weighted averaged vales (R^2^ ranges 0.58–0.76, Table 3, Fig. 4).

Stream temperature is strongly related to air temperature, and stream/air temperature are generally highly correlated at longer timeframes (such as monthly data, ref. 30). Plotting the average minimum and maximum water and sub-catchment air temperatures over the course of the year (Supplementary Fig. S2) shows how the observed stream temperature lies mostly between the minimum and maximum upstream air temperature. However, due to the heat capacity of streams, a lag-effect regarding the adaptation of stream to air temperatures can be observed (Supplementary Fig. S2 and ref. 30), such that for certain months, the air temperature in locations more distant (upstream average) or in the immediate upstream vicinity (distance-weighted average) may be better proxies of stream temperature, respectively. In this regard, high resolution data such as regional climate datasets can give more insights into these patters using e.g., the diurnal range of stream temperatures, and could be calculated using the newly created add-ons *r.stream.watersheds* and *r.stream.variables* along with a high-resolution stream network and regional climate datasets.

#### Monthly upstream precipitation versus observed monthly discharge

Monthly observed discharge data was downloaded from the Global River Discharge v.1.1 data set^31^ (Supplementary Fig. S1b). This data set contains monthly observed discharge in m^3^/sec from 1807–1991 at 1018 stations across the globe. Only observations after 1950 and from sites deemed reliable were used (flagged as such in the data set). We aggregated the monthly observations for each available year per station, where at least observations for three months needed to be available. A subset of 582 sites were marked reliable (71% of all stations) and were snapped to >=3rd order streams (3 km tolerance), since most gauging stations are located at larger rivers, but could be incorrectly snapped to smaller nearby confluences, leaving 568 sites for our validation (69%, Supplementary Fig. S1b). We then extracted the upstream sum and distance-weighted sum of the precipitation variables (Data Citation 1) at those locations.

Accounting for the upstream sum of precipitation gives a better estimate of the observed discharge, and R^2^ values ranged from 0.23 in August to 0.89 in March (Table 3, Fig. 5). It is therefore considered a more robust proxy for discharge than the distance weighted sum -metric, as the latter mentioned down weights the influence of precipitation at locations that are more distant to the discharge gauging station. Low goodness of fit scores of the upstream sum -metric are mostly due to low precipitation but high discharge patterns due to snow melt in winter/spring, and anthropogenic impact such as water management and release from reservoirs in arid regions during some months.

## Usage Notes

The newly developed variables (Data Citation 1) have a variety of potential uses in freshwater ecology, conservation and spatial biodiversity science. They are suited for applications the modelling and mapping of the spatial variation in freshwater species and communities. For example, the variables (Data Citation 1) can be used to annotate community data or point occurrences of freshwater species to explore their position in multivariate environmental niche space, to ascertain and predict the environmental limits to their distribution, or map their potential distribution. For modelling freshwater species distributions and most other use cases, we encourage choosing complementary variables from different variable categories to limit collinearity. An example of a useful combination may be the elevation *range* combined with the *average* upstream temperature, the upstream *sum* of precipitation, the *maximum* upstream land or soil cover and *weighted sum* of the surface geology cover. In certain cases, the upstream range and maximum value of a land cover variable can be more useful than the average, as anthropogenic influences (‘Urban/Built-up’) can depict the retention of land cover effects in downstream freshwater habitats rather than a dampening/uptake (which is indicated by the average and weighted average).

We provide the distance-weighted precipitation variables to users who wish to explore the precipitation patterns in the immediate vicinity of a given location, however the validation showed that they are not suitable as a proxy for discharge.

Further layers regarding future climate and land use projections are currently under development, and we encourage potential users to visit www.earthenv.org/streams for updates on the layers.

We provide example code in the Supplementary Information to load and process the variables in the netCDF format in R, and to use the GRASS add-ons ‘*r.stream.watersheds*’ and ‘*r.stream.variables*’.

## Additional Information

Table 2 is only available in the online version of this paper.

**How to cite this article:** Domisch, S. *et al.* Near-global freshwater-specific environmental variables for biodiversity analyses in 1 km resolution. *Sci. Data* 2:150073 doi: 10.1038/sdata.2015.73 (2015).

## Supplementary Material

Supplementary Information

## Figures and Tables

**Table 1:** Overview of the variable categories, the source and names of the variables, the number of source layers in each category, the unit of measurement, the naming convention of the netCDF files (Data Citation 1), and the metrics calculated for each variable

**Category**	**Source**	**Variable name**	**Number of source layers**	**Unit**	**Variable naming convention**	**Metric of upstream environment (number of layers)**						
						**Min**	**Max**	**Range**	**Average**	**Sum**	**Distance weighted average**	**Distance weighted sum**
Climate	WorldClim^6^	Minimum monthly air temperature	12	[°C] *10	monthly_tmin_*.nc	**12**	**12**	**12**	**12**	**−**	**12**	**−**
Climate	WorldClim^6^	Maximum monthly air temperature	12	[°C] *10	monthly_tmax_*.nc	**12**	**12**	**12**	**12**	**−**	**12**	**−**
Climate	WorldClim^6^	Monthly sum of precipitation	12	[mm]	monthly_prec_*.nc	**−**	**−**	**−**	**−**	**12**	**−**	**12**
Climate	WorldClim^6^	Long-term hydroclimatic variables	36	[°C] *10 and [mm]	hydroclim_*.nc	**−**	**−**	**−**	**11**	**8**	**11**	**8**
Topography	HydroSHEDS^12^	Elevation	1	[m]	elevation.nc	**1**	**1**	**1**	**1**	**−**	**−**	**−**
Topography	HydroSHEDS^12^	Slope	1	[°] * 100	slope.nc	**1**	**1**	**1**	**1**	**−**	**−**	**−**
Topography	HydroSHEDS^12^	Flow length (upstream cells)	1	count of grid cells	flow_acc.nc	**−**	**−**	**−**	**−**	**1**	**−**	**−**
Topography	HydroSHEDS^12^	Flow accumulation (watershed size)	1	count of grid cells	flow_acc.nc	**−**	**−**	**−**	**−**	**1**	**−**	**−**
Land cover	Consensus land-cover^8^	Land cover	12	Percent cover	landcover_*.nc	**12**	**12**	**12**	**12**	**−**	**12**	**−**
Surface geology	USGS^20^	Geological age	92	count of grid cells	geology_weighted_sum.nc	**−**	**−**	**−**	**−**	**−**	**−**	**92**
Soil	ISRIC ^21^	Soil type	10	%, cmol/kg, kg/m3, cm	soil_*.nc	**10**	**10**	**10**	**10**	**−**	**10**	**−**
Here, the numbers correspond to the number of calculated variables (Data Citation 1), the ‘−’ indicates that no variables were created for the given metric.												

**Table 2:** Full overview of all newly-developed freshwater-specific environmental variables

**Category**	**Type of upstream aggregation**	**netCDF file**	**Band ID**	**Variable code**	**Variable explanation**	**Unit**	**Datatype**	**NoData**	**Source**	**Native spatial grain**	**Data acquisition period**
Topography	minimum	elevation.nc	1	dem_min	Minimum elevation across sub-catchment	[m]	Int32	−999	HydroSHEDS	30 arcsec	2000
Topography	maximum	elevation.nc	2	dem_max	Maximum elevation across sub-catchment	[m]	Int32	−999	HydroSHEDS	30 arcsec	2000
Topography	range	elevation.nc	3	dem_range	Elevation range across sub-catchment	[m]	Int32	−999	HydroSHEDS	30 arcsec	2000
Topography	average	elevation.nc	4	dem_avg	Average elevation across sub-catchment	[m]	Int32	−999	HydroSHEDS	30 arcsec	2000
Topography	minimum	slope.nc	1	slope_min	Minimum slope across sub-catchment	[°] * 100	Int32	−999	HydroSHEDS	30 arcsec	2000
Topography	maximum	slope.nc	2	slope_max	Maximum slope across sub-catchment	[°] * 100	Int32	−999	HydroSHEDS	30 arcsec	2000
Topography	range	slope.nc	3	slope_range	Slope range across sub-catchment	[°] * 100	Int32	−999	HydroSHEDS	30 arcsec	2000
Topography	average	slope.nc	4	slope_avg	Average slope across sub-catchment	[°] * 100	Int32	−999	HydroSHEDS	30 arcsec	2000
Topography	sum	flow_acc.nc	1	flow_length	Flow length (number of upstream stream grid cells)	count	Int32	−999	HydroSHEDS	30 arcsec	2000
Topography	sum	flow_acc.nc	2	flow_acc	Flow accumulation (total number of upstream grid cells)	count	Int32	−999	HydroSHEDS	30 arcsec	2000
Climate	average	monthly_tmin_average.nc	1	tmin_avg_01	Average minimum monthly air temperature across water courses in sub-catchment for January	[°C] * 10	Int32	−999	WorldClim	30 arcsec	1950–2000
Climate	average	monthly_tmin_average.nc	2	tmin_avg_02	Average minimum monthly air temperature across water courses in sub-catchment for February	[°C] * 10	Int32	−999	WorldClim	30 arcsec	1950–2000
Climate	average	monthly_tmin_average.nc	3	tmin_avg_03	Average minimum monthly air temperature across water courses in sub-catchment for March	[°C] * 10	Int32	−999	WorldClim	30 arcsec	1950–2000
Climate	average	monthly_tmin_average.nc	4	tmin_avg_04	Average minimum monthly air temperature across water courses in sub-catchment for April	[°C] * 10	Int32	−999	WorldClim	30 arcsec	1950–2000
Climate	average	monthly_tmin_average.nc	5	tmin_avg_05	Average minimum monthly air temperature across water courses in sub-catchment for May	[°C] * 10	Int32	−999	WorldClim	30 arcsec	1950–2000
Climate	average	monthly_tmin_average.nc	6	tmin_avg_06	Average minimum monthly air temperature across water courses in sub-catchment for June	[°C] * 10	Int32	−999	WorldClim	30 arcsec	1950–2000
Climate	average	monthly_tmin_average.nc	7	tmin_avg_07	Average minimum monthly air temperature across water courses in sub-catchment for July	[°C] * 10	Int32	−999	WorldClim	30 arcsec	1950–2000
Climate	average	monthly_tmin_average.nc	8	tmin_avg_08	Average minimum monthly air temperature across water courses in sub-catchment for August	[°C] * 10	Int32	−999	WorldClim	30 arcsec	1950–2000
Climate	average	monthly_tmin_average.nc	9	tmin_avg_09	Average minimum monthly air temperature across water courses in sub-catchment for September	[°C] * 10	Int32	−999	WorldClim	30 arcsec	1950–2000
Climate	average	monthly_tmin_average.nc	10	tmin_avg_10	Average minimum monthly air temperature across water courses in sub-catchment for October	[°C] * 10	Int32	−999	WorldClim	30 arcsec	1950–2000
Climate	average	monthly_tmin_average.nc	11	tmin_avg_11	Average minimum monthly air temperature across water courses in sub-catchment for November	[°C] * 10	Int32	−999	WorldClim	30 arcsec	1950–2000
Climate	average	monthly_tmin_average.nc	12	tmin_avg_12	Average minimum monthly air temperature across water courses in sub-catchment for December	[°C] * 10	Int32	−999	WorldClim	30 arcsec	1950–2000
Climate	average	monthly_tmin_average.nc	1	tmax_avg_01	Average maximum monthly air temperature across water courses in sub-catchment for January	[°C] * 10	Int32	−999	WorldClim	30 arcsec	1950–2000
Climate	average	monthly_tmin_average.nc	2	tmax_avg_02	Average maximum monthly air temperature across water courses in sub-catchment for February	[°C] * 10	Int32	−999	WorldClim	30 arcsec	1950–2000
Climate	average	monthly_tmin_average.nc	3	tmax_avg_03	Average maximum monthly air temperature across water courses in sub-catchment for March	[°C] * 10	Int32	−999	WorldClim	30 arcsec	1950–2000
Climate	average	monthly_tmin_average.nc	4	tmax_avg_04	Average maximum monthly air temperature across water courses in sub-catchment for April	[°C] * 10	Int32	−999	WorldClim	30 arcsec	1950–2000
Climate	average	monthly_tmin_average.nc	5	tmax_avg_05	Average maximum monthly air temperature across water courses in sub-catchment for May	[°C] * 10	Int32	−999	WorldClim	30 arcsec	1950–2000
Climate	average	monthly_tmin_average.nc	6	tmax_avg_06	Average maximum monthly air temperature across water courses in sub-catchment for June	[°C] * 10	Int32	−999	WorldClim	30 arcsec	1950–2000
Climate	average	monthly_tmin_average.nc	7	tmax_avg_07	Average maximum monthly air temperature across water courses in sub-catchment for July	[°C] * 10	Int32	−999	WorldClim	30 arcsec	1950–2000
Climate	average	monthly_tmin_average.nc	8	tmax_avg_08	Average maximum monthly air temperature across water courses in sub-catchment for August	[°C] * 10	Int32	−999	WorldClim	30 arcsec	1950–2000
Climate	average	monthly_tmin_average.nc	9	tmax_avg_09	Average maximum monthly air temperature across water courses in sub-catchment for September	[°C] * 10	Int32	−999	WorldClim	30 arcsec	1950–2000
Climate	average	monthly_tmin_average.nc	10	tmax_avg_10	Average maximum monthly air temperature across water courses in sub-catchment for October	[°C] * 10	Int32	−999	WorldClim	30 arcsec	1950–2000
Climate	average	monthly_tmin_average.nc	11	tmax_avg_11	Average maximum monthly air temperature across water courses in sub-catchment for November	[°C] * 10	Int32	−999	WorldClim	30 arcsec	1950–2000
Climate	average	monthly_tmin_average.nc	12	tmax_avg_12	Average maximum monthly air temperature across water courses in sub-catchment for December	[°C] * 10	Int32	−999	WorldClim	30 arcsec	1950–2000
Climate	sum	monthly_prec_sum.nc	1	prec_sum_01	Sum of monthly precipitation across the sub-catchment for January	[mm]	Float64	−9999	WorldClim	30 arcsec	1950–2000
Climate	sum	monthly_prec_sum.nc	2	prec_sum_02	Sum of monthly precipitation across the sub-catchment for February	[mm]	Float64	−9999	WorldClim	30 arcsec	1950–2000
Climate	sum	monthly_prec_sum.nc	3	prec_sum_03	Sum of monthly precipitation across the sub-catchment for March	[mm]	Float64	−9999	WorldClim	30 arcsec	1950–2000
Climate	sum	monthly_prec_sum.nc	4	prec_sum_04	Sum of monthly precipitation across the sub-catchment for April	[mm]	Float64	−9999	WorldClim	30 arcsec	1950–2000
Climate	sum	monthly_prec_sum.nc	5	prec_sum_05	Sum of monthly precipitation across the sub-catchment for May	[mm]	Float64	−9999	WorldClim	30 arcsec	1950–2000
Climate	sum	monthly_prec_sum.nc	6	prec_sum_06	Sum of monthly precipitation across the sub-catchment for June	[mm]	Float64	−9999	WorldClim	30 arcsec	1950–2000
Climate	sum	monthly_prec_sum.nc	7	prec_sum_07	Sum of monthly precipitation across the sub-catchment for July	[mm]	Float64	−9999	WorldClim	30 arcsec	1950–2000
Climate	sum	monthly_prec_sum.nc	8	prec_sum_08	Sum of monthly precipitation across the sub-catchment for August	[mm]	Float64	−9999	WorldClim	30 arcsec	1950–2000
Climate	sum	monthly_prec_sum.nc	9	prec_sum_09	Sum of monthly precipitation across the sub-catchment for September	[mm]	Float64	−9999	WorldClim	30 arcsec	1950–2000
Climate	sum	monthly_prec_sum.nc	10	prec_sum_10	Sum of monthly precipitation across the sub-catchment for October	[mm]	Float64	−9999	WorldClim	30 arcsec	1950–2000
Climate	sum	monthly_prec_sum.nc	11	prec_sum_11	Sum of monthly precipitation across the sub-catchment for November	[mm]	Float64	−9999	WorldClim	30 arcsec	1950–2000
Climate	sum	monthly_prec_sum.nc	12	prec_sum_12	Sum of monthly precipitation across the sub-catchment for December	[mm]	Float64	−9999	WorldClim	30 arcsec	1950–2000
Climate	weighted average	monthly_tmin_average.nc	1	tmin_wavg_01	Weighted average minimum monthly air temperature across water courses in sub-catchment for January	[°C] * 10	Int32	−999	WorldClim	30 arcsec	1950–2000
Climate	weighted average	monthly_tmin_average.nc	2	tmin_wavg_02	Weighted average minimum monthly air temperature across water courses in sub-catchment for February	[°C] * 10	Int32	−999	WorldClim	30 arcsec	1950–2000
Climate	weighted average	monthly_tmin_average.nc	3	tmin_wavg_03	Weighted average minimum monthly air temperature across water courses in sub-catchment for March	[°C] * 10	Int32	−999	WorldClim	30 arcsec	1950–2000
Climate	weighted average	monthly_tmin_average.nc	4	tmin_wavg_04	Weighted average minimum monthly air temperature across water courses in sub-catchment for April	[°C] * 10	Int32	−999	WorldClim	30 arcsec	1950–2000
Climate	weighted average	monthly_tmin_average.nc	5	tmin_wavg_05	Weighted average minimum monthly air temperature across water courses in sub-catchment for May	[°C] * 10	Int32	−999	WorldClim	30 arcsec	1950–2000
Climate	weighted average	monthly_tmin_average.nc	6	tmin_wavg_06	Weighted average minimum monthly air temperature across water courses in sub-catchment for June	[°C] * 10	Int32	−999	WorldClim	30 arcsec	1950–2000
Climate	weighted average	monthly_tmin_average.nc	7	tmin_wavg_07	Weighted average minimum monthly air temperature across water courses in sub-catchment for July	[°C] * 10	Int32	−999	WorldClim	30 arcsec	1950–2000
Climate	weighted average	monthly_tmin_average.nc	8	tmin_wavg_08	Weighted average minimum monthly air temperature across water courses in sub-catchment for August	[°C] * 10	Int32	−999	WorldClim	30 arcsec	1950–2000
Climate	weighted average	monthly_tmin_average.nc	9	tmin_wavg_09	Weighted average minimum monthly air temperature across water courses in sub-catchment for September	[°C] * 10	Int32	−999	WorldClim	30 arcsec	1950–2000
Climate	weighted average	monthly_tmin_average.nc	10	tmin_wavg_10	Weighted average minimum monthly air temperature across water courses in sub-catchment for October	[°C] * 10	Int32	−999	WorldClim	30 arcsec	1950–2000
Climate	weighted average	monthly_tmin_average.nc	11	tmin_wavg_11	Weighted average minimum monthly air temperature across water courses in sub-catchment for November	[°C] * 10	Int32	−999	WorldClim	30 arcsec	1950–2000
Climate	weighted average	monthly_tmin_average.nc	12	tmin_wavg_12	Weighted average minimum monthly air temperature across water courses in sub-catchment for December	[°C] * 10	Int32	−999	WorldClim	30 arcsec	1950–2000
Climate	weighted average	monthly_tmin_average.nc	1	tmax_wavg_01	Weighted average maximum monthly air temperature across water courses in sub-catchment for January	[°C] * 10	Int32	−999	WorldClim	30 arcsec	1950–2000
Climate	weighted average	monthly_tmin_average.nc	2	tmax_wavg_02	Weighted average maximum monthly air temperature across water courses in sub-catchment for February	[°C] * 10	Int32	−999	WorldClim	30 arcsec	1950–2000
Climate	weighted average	monthly_tmin_average.nc	3	tmax_wavg_03	Weighted average maximum monthly air temperature across water courses in sub-catchment for March	[°C] * 10	Int32	−999	WorldClim	30 arcsec	1950–2000
Climate	weighted average	monthly_tmin_average.nc	4	tmax_wavg_04	Weighted average maximum monthly air temperature across water courses in sub-catchment for April	[°C] * 10	Int32	−999	WorldClim	30 arcsec	1950–2000
Climate	weighted average	monthly_tmin_average.nc	5	tmax_wavg_05	Weighted average maximum monthly air temperature across water courses in sub-catchment for May	[°C] * 10	Int32	−999	WorldClim	30 arcsec	1950–2000
Climate	weighted average	monthly_tmin_average.nc	6	tmax_wavg_06	Weighted average maximum monthly air temperature across water courses in sub-catchment for June	[°C] * 10	Int32	−999	WorldClim	30 arcsec	1950–2000
Climate	weighted average	monthly_tmin_average.nc	7	tmax_wavg_07	Weighted average maximum monthly air temperature across water courses in sub-catchment for July	[°C] * 10	Int32	−999	WorldClim	30 arcsec	1950–2000
Climate	weighted average	monthly_tmin_average.nc	8	tmax_wavg_08	Weighted average maximum monthly air temperature across water courses in sub-catchment for August	[°C] * 10	Int32	−999	WorldClim	30 arcsec	1950–2000
Climate	weighted average	monthly_tmin_average.nc	9	tmax_wavg_09	Weighted average maximum monthly air temperature across water courses in sub-catchment for September	[°C] * 10	Int32	−999	WorldClim	30 arcsec	1950–2000
Climate	weighted average	monthly_tmin_average.nc	10	tmax_wavg_10	Weighted average maximum monthly air temperature across water courses in sub-catchment for October	[°C] * 10	Int32	−999	WorldClim	30 arcsec	1950–2000
Climate	weighted average	monthly_tmin_average.nc	11	tmax_wavg_11	Weighted average maximum monthly air temperature across water courses in sub-catchment for November	[°C] * 10	Int32	−999	WorldClim	30 arcsec	1950–2000
Climate	weighted average	monthly_tmin_average.nc	12	tmax_wavg_12	Weighted average maximum monthly air temperature across water courses in sub-catchment for December	[°C] * 10	Int32	−999	WorldClim	30 arcsec	1950–2000
Climate	weighted sum	monthly_prec_sum.nc	1	prec_wsum_01	Weighted sum of monthly precipitation across the sub-catchment for January	[mm]	Float64	−9999	WorldClim	30 arcsec	1950–2000
Climate	weighted sum	monthly_prec_sum.nc	2	prec_wsum_02	Weighted sum of monthly precipitation across the sub-catchment for February	[mm]	Float64	−9999	WorldClim	30 arcsec	1950–2000
Climate	weighted sum	monthly_prec_sum.nc	3	prec_wsum_03	Weighted sum of monthly precipitation across the sub-catchment for March	[mm]	Float64	−9999	WorldClim	30 arcsec	1950–2000
Climate	weighted sum	monthly_prec_sum.nc	4	prec_wsum_04	Weighted sum of monthly precipitation across the sub-catchment for April	[mm]	Float64	−9999	WorldClim	30 arcsec	1950–2000
Climate	weighted sum	monthly_prec_sum.nc	5	prec_wsum_05	Weighted sum of monthly precipitation across the sub-catchment for May	[mm]	Float64	−9999	WorldClim	30 arcsec	1950–2000
Climate	weighted sum	monthly_prec_sum.nc	6	prec_wsum_06	Weighted sum of monthly precipitation across the sub-catchment for June	[mm]	Float64	−9999	WorldClim	30 arcsec	1950–2000
Climate	weighted sum	monthly_prec_sum.nc	7	prec_wsum_07	Weighted sum of monthly precipitation across the sub-catchment for July	[mm]	Float64	−9999	WorldClim	30 arcsec	1950–2000
Climate	weighted sum	monthly_prec_sum.nc	8	prec_wsum_08	Weighted sum of monthly precipitation across the sub-catchment for August	[mm]	Float64	−9999	WorldClim	30 arcsec	1950–2000
Climate	weighted sum	monthly_prec_sum.nc	9	prec_wsum_09	Weighted sum of monthly precipitation across the sub-catchment for September	[mm]	Float64	−9999	WorldClim	30 arcsec	1950–2000
Climate	weighted sum	monthly_prec_sum.nc	10	prec_wsum_10	Weighted sum of monthly precipitation across the sub-catchment for October	[mm]	Float64	−9999	WorldClim	30 arcsec	1950–2000
Climate	weighted sum	monthly_prec_sum.nc	11	prec_wsum_11	Weighted sum of monthly precipitation across the sub-catchment for November	[mm]	Float64	−9999	WorldClim	30 arcsec	1950–2000
Climate	weighted sum	monthly_prec_sum.nc	12	prec_wsum_12	Weighted sum of monthly precipitation across the sub-catchment for December	[mm]	Float64	−9999	WorldClim	30 arcsec	1950–2000
Climate	average	hydroclim_average+sum.nc	1	hydro_avg_01	Bioclim 1, average across sub-catchment (water courses only)—see http://worldclim.org/bioclim	[°C] * 10	Int32	−9999	WorldClim	30 arcsec	1950–2000
Climate	average	hydroclim_average+sum.nc	2	hydro_avg_02	Bioclim 2, average across sub-catchment (water courses only)—see http://worldclim.org/bioclim	[°C] * 10	Int32	−9999	WorldClim	30 arcsec	1950–2000
Climate	average	hydroclim_average+sum.nc	3	hydro_avg_03	Bioclim 3, average across sub-catchment (water courses only)—see http://worldclim.org/bioclim	* 100	Int32	−9999	WorldClim	30 arcsec	1950–2000
Climate	average	hydroclim_average+sum.nc	4	hydro_avg_04	Bioclim 4, average across sub-catchment (water courses only)—see http://worldclim.org/bioclim	[°C] * 10	Int32	−9999	WorldClim	30 arcsec	1950–2000
Climate	average	hydroclim_average+sum.nc	5	hydro_avg_05	Bioclim 5, average across sub-catchment (water courses only)—see http://worldclim.org/bioclim	[°C] * 10	Int32	−9999	WorldClim	30 arcsec	1950–2000
Climate	average	hydroclim_average+sum.nc	6	hydro_avg_06	Bioclim 6, average across sub-catchment (water courses only)—see http://worldclim.org/bioclim	[°C] * 10	Int32	−9999	WorldClim	30 arcsec	1950–2000
Climate	average	hydroclim_average+sum.nc	7	hydro_avg_07	Bioclim 7, average across sub-catchment (water courses only)—see http://worldclim.org/bioclim	[°C] * 10	Int32	−9999	WorldClim	30 arcsec	1950–2000
Climate	average	hydroclim_average+sum.nc	8	hydro_avg_08	Bioclim 8, average across sub-catchment (water courses only)—see http://worldclim.org/bioclim	[°C] * 10	Int32	−9999	WorldClim	30 arcsec	1950–2000
Climate	average	hydroclim_average+sum.nc	9	hydro_avg_09	Bioclim 9, average across sub-catchment (water courses only)—see http://worldclim.org/bioclim	[°C] * 10	Int32	−9999	WorldClim	30 arcsec	1950–2000
Climate	average	hydroclim_average+sum.nc	10	hydro_avg_10	Bioclim 10, average across sub-catchment (water courses only)—see http://worldclim.org/bioclim	[°C] * 10	Int32	−9999	WorldClim	30 arcsec	1950–2000
Climate	average	hydroclim_average+sum.nc	11	hydro_avg_11	Bioclim 11, average across sub-catchment (water courses only)—see http://worldclim.org/bioclim	[°C] * 10	Int32	−9999	WorldClim	30 arcsec	1950–2000
Climate	sum	hydroclim_average+sum.nc	12	hydro_avg_12	Bioclim 12, average across sub-catchment—see http://worldclim.org/bioclim	[mm]	Float64	−9999	WorldClim	30 arcsec	1950–2000
Climate	sum	hydroclim_average+sum.nc	13	hydro_avg_13	Bioclim 13, average across sub-catchment—see http://worldclim.org/bioclim	[mm]	Float64	−9999	WorldClim	30 arcsec	1950–2000
Climate	sum	hydroclim_average+sum.nc	14	hydro_avg_14	Bioclim 14, average across sub-catchment—see http://worldclim.org/bioclim	[mm]	Float64	−9999	WorldClim	30 arcsec	1950–2000
Climate	sum	hydroclim_average+sum.nc	15	hydro_avg_15	Bioclim 15, average across sub-catchment—see http://worldclim.org/bioclim	* 100	Float64	−9999	WorldClim	30 arcsec	1950–2000
Climate	sum	hydroclim_average+sum.nc	16	hydro_avg_16	Bioclim 16, average across sub-catchment—see http://worldclim.org/bioclim	[mm]	Float64	−9999	WorldClim	30 arcsec	1950–2000
Climate	sum	hydroclim_average+sum.nc	17	hydro_avg_17	Bioclim 17, average across sub-catchment—see http://worldclim.org/bioclim	[mm]	Float64	−9999	WorldClim	30 arcsec	1950–2000
Climate	sum	hydroclim_average+sum.nc	18	hydro_avg_18	Bioclim 18, average across sub-catchment—see http://worldclim.org/bioclim	[mm]	Float64	−9999	WorldClim	30 arcsec	1950–2000
Climate	sum	hydroclim_average+sum.nc	19	hydro_avg_19	Bioclim 19, average across sub-catchment—see http://worldclim.org/bioclim	[mm]	Float64	−9999	WorldClim	30 arcsec	1950–2000
Climate	weighted average	hydroclim_weighted_average+sum.nc	1	hydro_wavg_01	Bioclim 1, weighted average across sub-catchment (water courses only)—see http://worldclim.org/bioclim	[°C] * 10	Int32	−9999	WorldClim	30 arcsec	1950–2000
Climate	weighted average	hydroclim_weighted_average+sum.nc	2	hydro_wavg_02	Bioclim 2, weighted average across sub-catchment (water courses only)—see http://worldclim.org/bioclim	[°C] * 10	Int32	−9999	WorldClim	30 arcsec	1950–2000
Climate	weighted average	hydroclim_weighted_average+sum.nc	3	hydro_wavg_03	Bioclim 3, weighted average across sub-catchment (water courses only)—see http://worldclim.org/bioclim	* 100	Int32	−9999	WorldClim	30 arcsec	1950–2000
Climate	weighted average	hydroclim_weighted_average+sum.nc	4	hydro_wavg_04	Bioclim 4, weighted average across sub-catchment (water courses only)—see http://worldclim.org/bioclim	[°C] * 10	Int32	−9999	WorldClim	30 arcsec	1950–2000
Climate	weighted average	hydroclim_weighted_average+sum.nc	5	hydro_wavg_05	Bioclim 5, weighted average across sub-catchment (water courses only)—see http://worldclim.org/bioclim	[°C] * 10	Int32	−9999	WorldClim	30 arcsec	1950–2000
Climate	weighted average	hydroclim_weighted_average+sum.nc	6	hydro_wavg_06	Bioclim 6, weighted average across sub-catchment (water courses only)—see http://worldclim.org/bioclim	[°C] * 10	Int32	−9999	WorldClim	30 arcsec	1950–2000
Climate	weighted average	hydroclim_weighted_average+sum.nc	7	hydro_wavg_07	Bioclim 7, weighted average across sub-catchment (water courses only)—see http://worldclim.org/bioclim	[°C] * 10	Int32	−9999	WorldClim	30 arcsec	1950–2000
Climate	weighted average	hydroclim_weighted_average+sum.nc	8	hydro_wavg_08	Bioclim 8, weighted average across sub-catchment (water courses only)—see http://worldclim.org/bioclim	[°C] * 10	Int32	−9999	WorldClim	30 arcsec	1950–2000
Climate	weighted average	hydroclim_weighted_average+sum.nc	9	hydro_wavg_09	Bioclim 9, weighted average across sub-catchment (water courses only)—see http://worldclim.org/bioclim	[°C] * 10	Int32	−9999	WorldClim	30 arcsec	1950–2000
Climate	weighted average	hydroclim_weighted_average+sum.nc	10	hydro_wavg_10	Bioclim 10, weighted average across sub-catchment (water courses only)—see http://worldclim.org/bioclim	[°C] * 10	Int32	−9999	WorldClim	30 arcsec	1950–2000
Climate	weighted average	hydroclim_weighted_average+sum.nc	11	hydro_wavg_11	Bioclim 11, weighted average across sub-catchment (water courses only)—see http://worldclim.org/bioclim	[°C] * 10	Int32	−9999	WorldClim	30 arcsec	1950–2000
Climate	weighted sum	hydroclim_weighted_average+sum.nc	12	hydro_wavg_12	Bioclim 12, weighted average across sub-catchment—see http://worldclim.org/bioclim	[mm]	Float64	−9999	WorldClim	30 arcsec	1950–2000
Climate	weighted sum	hydroclim_weighted_average+sum.nc	13	hydro_wavg_13	Bioclim 13, weighted average across sub-catchment—see http://worldclim.org/bioclim	[mm]	Float64	−9999	WorldClim	30 arcsec	1950–2000
Climate	weighted sum	hydroclim_weighted_average+sum.nc	14	hydro_wavg_14	Bioclim 14, weighted average across sub-catchment—see http://worldclim.org/bioclim	[mm]	Float64	−9999	WorldClim	30 arcsec	1950–2000
Climate	weighted sum	hydroclim_weighted_average+sum.nc	15	hydro_wavg_15	Bioclim 15, weighted average across sub-catchment—see http://worldclim.org/bioclim	* 100	Float64	−9999	WorldClim	30 arcsec	1950–2000
Climate	weighted sum	hydroclim_weighted_average+sum.nc	16	hydro_wavg_16	Bioclim 16, weighted average across sub-catchment—see http://worldclim.org/bioclim	[mm]	Float64	−9999	WorldClim	30 arcsec	1950–2000
Climate	weighted sum	hydroclim_weighted_average+sum.nc	17	hydro_wavg_17	Bioclim 17, weighted average across sub-catchment—see http://worldclim.org/bioclim	[mm]	Float64	−9999	WorldClim	30 arcsec	1950–2000
Climate	weighted sum	hydroclim_weighted_average+sum.nc	18	hydro_wavg_18	Bioclim 18, weighted average across sub-catchment—see http://worldclim.org/bioclim	[mm]	Float64	−9999	WorldClim	30 arcsec	1950–2000
Climate	weighted sum	hydroclim_weighted_average+sum.nc	19	hydro_wavg_19	Bioclim 19, weighted average across sub-catchment—see http://worldclim.org/bioclim	[mm]	Float64	−9999	WorldClim	30 arcsec	1950–2000
Land cover	minimum	landcover_minimum.nc	1	lc_min_01	Evergreen/deciduous needleleaf trees, minimum across sub-catchment	[%]	Byte	−127	Consensus land cover	30 arcsec	1992–2006
Land cover	minimum	landcover_minimum.nc	2	lc_min_02	Evergreen broadleaf trees, minimum across sub-catchment	[%]	Byte	−127	Consensus land cover	30 arcsec	1992–2006
Land cover	minimum	landcover_minimum.nc	3	lc_min_03	Deciduous broadleaf trees, minimum across sub-catchment	[%]	Byte	−127	Consensus land cover	30 arcsec	1992–2006
Land cover	minimum	landcover_minimum.nc	4	lc_min_04	Mixed/other trees, minimum across sub-catchment	[%]	Byte	−127	Consensus land cover	30 arcsec	1992–2006
Land cover	minimum	landcover_minimum.nc	5	lc_min_05	Shrubs, minimum across sub-catchment	[%]	Byte	−127	Consensus land cover	30 arcsec	1992–2006
Land cover	minimum	landcover_minimum.nc	6	lc_min_06	Herbaceous vegetation, minimum across sub-catchment	[%]	Byte	−127	Consensus land cover	30 arcsec	1992–2006
Land cover	minimum	landcover_minimum.nc	7	lc_min_07	Cultivated and managed vegetation, minimum across sub-catchment	[%]	Byte	−127	Consensus land cover	30 arcsec	1992–2006
Land cover	minimum	landcover_minimum.nc	8	lc_min_08	Regularly flooded shrub/herbaceous vegetation, minimum across sub-catchment	[%]	Byte	−127	Consensus land cover	30 arcsec	1992–2006
Land cover	minimum	landcover_minimum.nc	9	lc_min_09	Urban/built-up, minimum across sub-catchment	[%]	Byte	−127	Consensus land cover	30 arcsec	1992–2006
Land cover	minimum	landcover_minimum.nc	10	lc_min_10	Snow/ice, minimum across sub-catchment	[%]	Byte	−127	Consensus land cover	30 arcsec	1992–2006
Land cover	minimum	landcover_minimum.nc	11	lc_min_11	Barren lands/sparse vegetation, minimum across sub-catchment	[%]	Byte	−127	Consensus land cover	30 arcsec	1992–2006
Land cover	minimum	landcover_minimum.nc	12	lc_min_12	Open water, minimum across sub-catchment	[%]	Byte	−127	Consensus land cover	30 arcsec	1992–2006
Land cover	maximum	landcover_maximum.nc	1	lc_max_01	Evergreen/deciduous needleleaf trees, maximum across sub-catchment	[%]	Byte	−127	Consensus land cover	30 arcsec	1992–2006
Land cover	maximum	landcover_maximum.nc	2	lc_max_02	Evergreen broadleaf trees, maximum across sub-catchment	[%]	Byte	−127	Consensus land cover	30 arcsec	1992–2006
Land cover	maximum	landcover_maximum.nc	3	lc_max_03	Deciduous broadleaf trees, maximum across sub-catchment	[%]	Byte	−127	Consensus land cover	30 arcsec	1992–2006
Land cover	maximum	landcover_maximum.nc	4	lc_max_04	Mixed/other trees, maximum across sub-catchment	[%]	Byte	−127	Consensus land cover	30 arcsec	1992–2006
Land cover	maximum	landcover_maximum.nc	5	lc_max_05	Shrubs, maximum across sub-catchment	[%]	Byte	−127	Consensus land cover	30 arcsec	1992–2006
Land cover	maximum	landcover_maximum.nc	6	lc_max_06	Herbaceous vegetation, maximum across sub-catchment	[%]	Byte	−127	Consensus land cover	30 arcsec	1992–2006
Land cover	maximum	landcover_maximum.nc	7	lc_max_07	Cultivated and managed vegetation, maximum across sub-catchment	[%]	Byte	−127	Consensus land cover	30 arcsec	1992–2006
Land cover	maximum	landcover_maximum.nc	8	lc_max_08	Regularly flooded shrub/herbaceous vegetation, maximum across sub-catchment	[%]	Byte	−127	Consensus land cover	30 arcsec	1992–2006
Land cover	maximum	landcover_maximum.nc	9	lc_max_09	Urban/built-up, maximum across sub-catchment	[%]	Byte	−127	Consensus land cover	30 arcsec	1992–2006
Land cover	maximum	landcover_maximum.nc	10	lc_max_10	Snow/ice, maximum across sub-catchment	[%]	Byte	−127	Consensus land cover	30 arcsec	1992–2006
Land cover	maximum	landcover_maximum.nc	11	lc_max_11	Barren lands/sparse vegetation, maximum across sub-catchment	[%]	Byte	−127	Consensus land cover	30 arcsec	1992–2006
Land cover	maximum	landcover_maximum.nc	12	lc_max_12	Open water, maximum across sub-catchment	[%]	Byte	−127	Consensus land cover	30 arcsec	1992–2006
Land cover	range	landcover_range.nc	1	lc_range_01	Evergreen/deciduous needleleaf trees, range across sub-catchment	[%]	Byte	−127	Consensus land cover	30 arcsec	1992–2006
Land cover	range	landcover_range.nc	2	lc_range_02	Evergreen broadleaf trees, range across sub-catchment	[%]	Byte	−127	Consensus land cover	30 arcsec	1992–2006
Land cover	range	landcover_range.nc	3	lc_range_03	Deciduous broadleaf trees, range across sub-catchment	[%]	Byte	−127	Consensus land cover	30 arcsec	1992–2006
Land cover	range	landcover_range.nc	4	lc_range_04	Mixed/other trees, range across sub-catchment	[%]	Byte	−127	Consensus land cover	30 arcsec	1992–2006
Land cover	range	landcover_range.nc	5	lc_range_05	Shrubs, range across sub-catchment	[%]	Byte	−127	Consensus land cover	30 arcsec	1992–2006
Land cover	range	landcover_range.nc	6	lc_range_06	Herbaceous vegetation, range across sub-catchment	[%]	Byte	−127	Consensus land cover	30 arcsec	1992–2006
Land cover	range	landcover_range.nc	7	lc_range_07	Cultivated and managed vegetation, range across sub-catchment	[%]	Byte	−127	Consensus land cover	30 arcsec	1992–2006
Land cover	range	landcover_range.nc	8	lc_range_08	Regularly flooded shrub/herbaceous vegetation, range across sub-catchment	[%]	Byte	−127	Consensus land cover	30 arcsec	1992–2006
Land cover	range	landcover_range.nc	9	lc_range_09	Urban/built-up, range across sub-catchment	[%]	Byte	−127	Consensus land cover	30 arcsec	1992–2006
Land cover	range	landcover_range.nc	10	lc_range_10	Snow/ice, range across sub-catchment	[%]	Byte	−127	Consensus land cover	30 arcsec	1992–2006
Land cover	range	landcover_range.nc	11	lc_range_11	Barren lands/sparse vegetation, range across sub-catchment	[%]	Byte	−127	Consensus land cover	30 arcsec	1992–2006
Land cover	range	landcover_range.nc	12	lc_range_12	Open water, range across sub-catchment	[%]	Byte	−127	Consensus land cover	30 arcsec	1992–2006
Land cover	average	landcover_average.nc	1	lc_avg_01	Evergreen/deciduous needleleaf trees, average across sub-catchment	[%]	Byte	−127	Consensus land cover	30 arcsec	1992–2006
Land cover	average	landcover_average.nc	2	lc_avg_02	Evergreen broadleaf trees, average across sub-catchment	[%]	Byte	−127	Consensus land cover	30 arcsec	1992–2006
Land cover	average	landcover_average.nc	3	lc_avg_03	Deciduous broadleaf trees, average across sub-catchment	[%]	Byte	−127	Consensus land cover	30 arcsec	1992–2006
Land cover	average	landcover_average.nc	4	lc_avg_04	Mixed/other trees, average across sub-catchment	[%]	Byte	−127	Consensus land cover	30 arcsec	1992–2006
Land cover	average	landcover_average.nc	5	lc_avg_05	Shrubs, average across sub-catchment	[%]	Byte	−127	Consensus land cover	30 arcsec	1992–2006
Land cover	average	landcover_average.nc	6	lc_avg_06	Herbaceous vegetation, average across sub-catchment	[%]	Byte	−127	Consensus land cover	30 arcsec	1992–2006
Land cover	average	landcover_average.nc	7	lc_avg_07	Cultivated and managed vegetation, average across sub-catchment	[%]	Byte	−127	Consensus land cover	30 arcsec	1992–2006
Land cover	average	landcover_average.nc	8	lc_avg_08	Regularly flooded shrub/herbaceous vegetation, average across sub-catchment	[%]	Byte	−127	Consensus land cover	30 arcsec	1992–2006
Land cover	average	landcover_average.nc	9	lc_avg_09	Urban/built-up, average across sub-catchment	[%]	Byte	−127	Consensus land cover	30 arcsec	1992–2006
Land cover	average	landcover_average.nc	10	lc_avg_10	Snow/ice, average across sub-catchment	[%]	Byte	−127	Consensus land cover	30 arcsec	1992–2006
Land cover	average	landcover_average.nc	11	lc_avg_11	Barren lands/sparse vegetation, average across sub-catchment	[%]	Byte	−127	Consensus land cover	30 arcsec	1992–2006
Land cover	average	landcover_average.nc	12	lc_avg_12	Open water, average across sub-catchment	[%]	Byte	−127	Consensus land cover	30 arcsec	1992–2006
Land cover	weighted average	landcover_weighted_average.nc	1	lc_wavg_01	Weighted average land cover across sub-catchment: Evergreen/deciduous needleleaf trees	[%]	Byte	−127	Consensus land cover	30 arcsec	1992–2006
Land cover	weighted average	landcover_weighted_average.nc	2	lc_wavg_02	Weighted average land cover across sub-catchment: Evergreen broadleaf trees	[%]	Byte	−127	Consensus land cover	30 arcsec	1992–2006
Land cover	weighted average	landcover_weighted_average.nc	3	lc_wavg_03	Weighted average land cover across sub-catchment: Deciduous broadleaf trees	[%]	Byte	−127	Consensus land cover	30 arcsec	1992–2006
Land cover	weighted average	landcover_weighted_average.nc	4	lc_wavg_04	Weighted average land cover across sub-catchment: Mixed/other trees	[%]	Byte	−127	Consensus land cover	30 arcsec	1992–2006
Land cover	weighted average	landcover_weighted_average.nc	5	lc_wavg_05	Weighted average land cover across sub-catchment: Shrubs	[%]	Byte	−127	Consensus land cover	30 arcsec	1992–2006
Land cover	weighted average	landcover_weighted_average.nc	6	lc_wavg_06	Weighted average land cover across sub-catchment: Herbaceous vegetation	[%]	Byte	−127	Consensus land cover	30 arcsec	1992–2006
Land cover	weighted average	landcover_weighted_average.nc	7	lc_wavg_07	Weighted average land cover across sub-catchment: Cultivated and managed vegetation	[%]	Byte	−127	Consensus land cover	30 arcsec	1992–2006
Land cover	weighted average	landcover_weighted_average.nc	8	lc_wavg_08	Weighted average land cover across sub-catchment: Regularly flooded shrub/herbaceous vegetation	[%]	Byte	−127	Consensus land cover	30 arcsec	1992–2006
Land cover	weighted average	landcover_weighted_average.nc	9	lc_wavg_09	Weighted average land cover across sub-catchment: Urban/built-up	[%]	Byte	−127	Consensus land cover	30 arcsec	1992–2006
Land cover	weighted average	landcover_weighted_average.nc	10	lc_wavg_10	Weighted average land cover across sub-catchment: Snow/ice	[%]	Byte	−127	Consensus land cover	30 arcsec	1992–2006
Land cover	weighted average	landcover_weighted_average.nc	11	lc_wavg_11	Weighted average land cover across sub-catchment: Barren lands/sparse vegetation	[%]	Byte	−127	Consensus land cover	30 arcsec	1992–2006
Land cover	weighted average	landcover_weighted_average.nc	12	lc_wavg_12	Weighted average land cover across sub-catchment: Open water	[%]	Byte	−127	Consensus land cover	30 arcsec	1992–2006
Surface geology	weighted sum	geology_weighted_sum.nc	1	geo_wsum_01	Archean geology across sub-catchment	weighted count	Int32	−9999	USGS	30 arcsec	1960–2000
Surface geology	weighted sum	geology_weighted_sum.nc	2	geo_wsum_02	Archean, Permian geology across sub-catchment	weighted count	Int32	−9999	USGS	30 arcsec	1960–2000
Surface geology	weighted sum	geology_weighted_sum.nc	3	geo_wsum_03	Cambrian geology across sub-catchment	weighted count	Int32	−9999	USGS	30 arcsec	1960–2000
Surface geology	weighted sum	geology_weighted_sum.nc	4	geo_wsum_04	Cambrian, Precambrian geology across sub-catchment	weighted count	Int32	−9999	USGS	30 arcsec	1960–2000
Surface geology	weighted sum	geology_weighted_sum.nc	5	geo_wsum_05	Cambrian, Proterozoic geology across sub-catchment	weighted count	Int32	−9999	USGS	30 arcsec	1960–2000
Surface geology	weighted sum	geology_weighted_sum.nc	6	geo_wsum_06	Carboniferous geology across sub-catchment	weighted count	Int32	−9999	USGS	30 arcsec	1960–2000
Surface geology	weighted sum	geology_weighted_sum.nc	7	geo_wsum_07	Carboniferous, Devonian geology across sub-catchment	weighted count	Int32	−9999	USGS	30 arcsec	1960–2000
Surface geology	weighted sum	geology_weighted_sum.nc	8	geo_wsum_08	Carboniferous, Miocene geology across sub-catchment	weighted count	Int32	−9999	USGS	30 arcsec	1960–2000
Surface geology	weighted sum	geology_weighted_sum.nc	9	geo_wsum_09	Cenozoic geology across sub-catchment	weighted count	Int32	−9999	USGS	30 arcsec	1960–2000
Surface geology	weighted sum	geology_weighted_sum.nc	10	geo_wsum_10	Cenozoic, Mesozoic geology across sub-catchment	weighted count	Int32	−9999	USGS	30 arcsec	1960–2000
Surface geology	weighted sum	geology_weighted_sum.nc	11	geo_wsum_11	Cretaceous geology across sub-catchment	weighted count	Int32	−9999	USGS	30 arcsec	1960–2000
Surface geology	weighted sum	geology_weighted_sum.nc	12	geo_wsum_12	Cretaceous, Carboniferous geology across sub-catchment	weighted count	Int32	−9999	USGS	30 arcsec	1960–2000
Surface geology	weighted sum	geology_weighted_sum.nc	13	geo_wsum_13	Cretaceous, Devonian geology across sub-catchment	weighted count	Int32	−9999	USGS	30 arcsec	1960–2000
Surface geology	weighted sum	geology_weighted_sum.nc	14	geo_wsum_14	Cretaceous, Jurassic geology across sub-catchment	weighted count	Int32	−9999	USGS	30 arcsec	1960–2000
Surface geology	weighted sum	geology_weighted_sum.nc	15	geo_wsum_15	Cretaceous, Mississippian geology across sub-catchment	weighted count	Int32	−9999	USGS	30 arcsec	1960–2000
Surface geology	weighted sum	geology_weighted_sum.nc	16	geo_wsum_16	Cretaceous, Paleogene, Neogene geology across sub-catchment	weighted count	Int32	−9999	USGS	30 arcsec	1960–2000
Surface geology	weighted sum	geology_weighted_sum.nc	17	geo_wsum_17	Cretaceous, Permian geology across sub-catchment	weighted count	Int32	−9999	USGS	30 arcsec	1960–2000
Surface geology	weighted sum	geology_weighted_sum.nc	18	geo_wsum_18	Cretaceous, Tertiary geology across sub-catchment	weighted count	Int32	−9999	USGS	30 arcsec	1960–2000
Surface geology	weighted sum	geology_weighted_sum.nc	19	geo_wsum_19	Cretaceous, Triassic geology across sub-catchment	weighted count	Int32	−9999	USGS	30 arcsec	1960–2000
Surface geology	weighted sum	geology_weighted_sum.nc	20	geo_wsum_20	Devonian geology across sub-catchment	weighted count	Int32	−9999	USGS	30 arcsec	1960–2000
Surface geology	weighted sum	geology_weighted_sum.nc	21	geo_wsum_21	Devonian, Cambrian geology across sub-catchment	weighted count	Int32	−9999	USGS	30 arcsec	1960–2000
Surface geology	weighted sum	geology_weighted_sum.nc	22	geo_wsum_22	Devonian, Ordovician geology across sub-catchment	weighted count	Int32	−9999	USGS	30 arcsec	1960–2000
Surface geology	weighted sum	geology_weighted_sum.nc	23	geo_wsum_23	Devonian, Proterozoic geology across sub-catchment	weighted count	Int32	−9999	USGS	30 arcsec	1960–2000
Surface geology	weighted sum	geology_weighted_sum.nc	24	geo_wsum_24	Devonian, Silurian geology across sub-catchment	weighted count	Int32	−9999	USGS	30 arcsec	1960–2000
Surface geology	weighted sum	geology_weighted_sum.nc	25	geo_wsum_25	Devonian, Silurian, Ordovician geology across sub-catchment	weighted count	Int32	−9999	USGS	30 arcsec	1960–2000
Surface geology	weighted sum	geology_weighted_sum.nc	26	geo_wsum_26	Holocene geology across sub-catchment	weighted count	Int32	−9999	USGS	30 arcsec	1960–2000
Surface geology	weighted sum	geology_weighted_sum.nc	27	geo_wsum_27	Ice geology across sub-catchment	weighted count	Int32	−9999	USGS	30 arcsec	1960–2000
Surface geology	weighted sum	geology_weighted_sum.nc	28	geo_wsum_28	Jurassic geology across sub-catchment	weighted count	Int32	−9999	USGS	30 arcsec	1960–2000
Surface geology	weighted sum	geology_weighted_sum.nc	29	geo_wsum_29	Jurassic, Cambrian geology across sub-catchment	weighted count	Int32	−9999	USGS	30 arcsec	1960–2000
Surface geology	weighted sum	geology_weighted_sum.nc	30	geo_wsum_30	Jurassic, Carboniferous geology across sub-catchment	weighted count	Int32	−9999	USGS	30 arcsec	1960–2000
Surface geology	weighted sum	geology_weighted_sum.nc	31	geo_wsum_31	Jurassic, Devonian geology across sub-catchment	weighted count	Int32	−9999	USGS	30 arcsec	1960–2000
Surface geology	weighted sum	geology_weighted_sum.nc	32	geo_wsum_32	Jurassic, Mississippian geology across sub-catchment	weighted count	Int32	−9999	USGS	30 arcsec	1960–2000
Surface geology	weighted sum	geology_weighted_sum.nc	33	geo_wsum_33	Jurassic, Ordovician geology across sub-catchment	weighted count	Int32	−9999	USGS	30 arcsec	1960–2000
Surface geology	weighted sum	geology_weighted_sum.nc	34	geo_wsum_34	Jurassic, Permian geology across sub-catchment	weighted count	Int32	−9999	USGS	30 arcsec	1960–2000
Surface geology	weighted sum	geology_weighted_sum.nc	35	geo_wsum_35	Jurassic, Triassic geology across sub-catchment	weighted count	Int32	−9999	USGS	30 arcsec	1960–2000
Surface geology	weighted sum	geology_weighted_sum.nc	36	geo_wsum_36	Kimberlite geology across sub-catchment	weighted count	Int32	−9999	USGS	30 arcsec	1960–2000
Surface geology	weighted sum	geology_weighted_sum.nc	37	geo_wsum_37	Mesozoic geology across sub-catchment	weighted count	Int32	−9999	USGS	30 arcsec	1960–2000
Surface geology	weighted sum	geology_weighted_sum.nc	38	geo_wsum_38	Mesozoic, Cenozoic geology across sub-catchment	weighted count	Int32	−9999	USGS	30 arcsec	1960–2000
Surface geology	weighted sum	geology_weighted_sum.nc	39	geo_wsum_39	Mesozoic, Paleozoic geology across sub-catchment	weighted count	Int32	−9999	USGS	30 arcsec	1960–2000
Surface geology	weighted sum	geology_weighted_sum.nc	40	geo_wsum_40	Mesozoic, Palezoic geology across sub-catchment	weighted count	Int32	−9999	USGS	30 arcsec	1960–2000
Surface geology	weighted sum	geology_weighted_sum.nc	41	geo_wsum_41	Miocene geology across sub-catchment	weighted count	Int32	−9999	USGS	30 arcsec	1960–2000
Surface geology	weighted sum	geology_weighted_sum.nc	42	geo_wsum_42	Mississippian geology across sub-catchment	weighted count	Int32	−9999	USGS	30 arcsec	1960–2000
Surface geology	weighted sum	geology_weighted_sum.nc	43	geo_wsum_43	Mississippian, Cambrian geology across sub-catchment	weighted count	Int32	−9999	USGS	30 arcsec	1960–2000
Surface geology	weighted sum	geology_weighted_sum.nc	44	geo_wsum_44	Mississippian, Devonian geology across sub-catchment	weighted count	Int32	−9999	USGS	30 arcsec	1960–2000
Surface geology	weighted sum	geology_weighted_sum.nc	45	geo_wsum_45	Neogene geology across sub-catchment	weighted count	Int32	−9999	USGS	30 arcsec	1960–2000
Surface geology	weighted sum	geology_weighted_sum.nc	46	geo_wsum_46	Neogene, Paleogene geology across sub-catchment	weighted count	Int32	−9999	USGS	30 arcsec	1960–2000
Surface geology	weighted sum	geology_weighted_sum.nc	47	geo_wsum_47	Ordovician geology across sub-catchment	weighted count	Int32	−9999	USGS	30 arcsec	1960–2000
Surface geology	weighted sum	geology_weighted_sum.nc	48	geo_wsum_48	Ordovician, Cambrian geology across sub-catchment	weighted count	Int32	−9999	USGS	30 arcsec	1960–2000
Surface geology	weighted sum	geology_weighted_sum.nc	49	geo_wsum_49	Ordovician, Proterozoic geology across sub-catchment	weighted count	Int32	−9999	USGS	30 arcsec	1960–2000
Surface geology	weighted sum	geology_weighted_sum.nc	50	geo_wsum_50	Paleogene geology across sub-catchment	weighted count	Int32	−9999	USGS	30 arcsec	1960–2000
Surface geology	weighted sum	geology_weighted_sum.nc	51	geo_wsum_51	Paleogene, Cretaceous geology across sub-catchment	weighted count	Int32	−9999	USGS	30 arcsec	1960–2000
Surface geology	weighted sum	geology_weighted_sum.nc	52	geo_wsum_52	Paleozoic geology across sub-catchment	weighted count	Int32	−9999	USGS	30 arcsec	1960–2000
Surface geology	weighted sum	geology_weighted_sum.nc	53	geo_wsum_53	Paleozoic, Mesozoic geology across sub-catchment	weighted count	Int32	−9999	USGS	30 arcsec	1960–2000
Surface geology	weighted sum	geology_weighted_sum.nc	54	geo_wsum_54	Paleozoic, Precambrian geology across sub-catchment	weighted count	Int32	−9999	USGS	30 arcsec	1960–2000
Surface geology	weighted sum	geology_weighted_sum.nc	55	geo_wsum_55	Paleozoic, Proterozoic geology across sub-catchment	weighted count	Int32	−9999	USGS	30 arcsec	1960–2000
Surface geology	weighted sum	geology_weighted_sum.nc	56	geo_wsum_56	Pennsylvanian geology across sub-catchment	weighted count	Int32	−9999	USGS	30 arcsec	1960–2000
Surface geology	weighted sum	geology_weighted_sum.nc	57	geo_wsum_57	Pennsylvanian, Devonian geology across sub-catchment	weighted count	Int32	−9999	USGS	30 arcsec	1960–2000
Surface geology	weighted sum	geology_weighted_sum.nc	58	geo_wsum_58	Pennsylvanian, Mississippian geology across sub-catchment	weighted count	Int32	−9999	USGS	30 arcsec	1960–2000
Surface geology	weighted sum	geology_weighted_sum.nc	59	geo_wsum_59	Permian geology across sub-catchment	weighted count	Int32	−9999	USGS	30 arcsec	1960–2000
Surface geology	weighted sum	geology_weighted_sum.nc	60	geo_wsum_60	Permian, Carboniferous geology across sub-catchment	weighted count	Int32	−9999	USGS	30 arcsec	1960–2000
Surface geology	weighted sum	geology_weighted_sum.nc	61	geo_wsum_61	Permian, Devonian geology across sub-catchment	weighted count	Int32	−9999	USGS	30 arcsec	1960–2000
Surface geology	weighted sum	geology_weighted_sum.nc	62	geo_wsum_62	Permian, Mississippian geology across sub-catchment	weighted count	Int32	−9999	USGS	30 arcsec	1960–2000
Surface geology	weighted sum	geology_weighted_sum.nc	63	geo_wsum_63	Permian, Pennsylvanian geology across sub-catchment	weighted count	Int32	−9999	USGS	30 arcsec	1960–2000
Surface geology	weighted sum	geology_weighted_sum.nc	64	geo_wsum_64	Permian, Triassic geology across sub-catchment	weighted count	Int32	−9999	USGS	30 arcsec	1960–2000
Surface geology	weighted sum	geology_weighted_sum.nc	65	geo_wsum_65	Pleistocene geology across sub-catchment	weighted count	Int32	−9999	USGS	30 arcsec	1960–2000
Surface geology	weighted sum	geology_weighted_sum.nc	66	geo_wsum_66	Pliocene, Quaternary geology across sub-catchment	weighted count	Int32	−9999	USGS	30 arcsec	1960–2000
Surface geology	weighted sum	geology_weighted_sum.nc	67	geo_wsum_67	Precambrian geology across sub-catchment	weighted count	Int32	−9999	USGS	30 arcsec	1960–2000
Surface geology	weighted sum	geology_weighted_sum.nc	68	geo_wsum_68	Precambrian, Devonian geology across sub-catchment	weighted count	Int32	−9999	USGS	30 arcsec	1960–2000
Surface geology	weighted sum	geology_weighted_sum.nc	69	geo_wsum_69	Precambrian, Paleozoic geology across sub-catchment	weighted count	Int32	−9999	USGS	30 arcsec	1960–2000
Surface geology	weighted sum	geology_weighted_sum.nc	70	geo_wsum_70	Proterozoic geology across sub-catchment	weighted count	Int32	−9999	USGS	30 arcsec	1960–2000
Surface geology	weighted sum	geology_weighted_sum.nc	71	geo_wsum_71	Proterozoic, Archean geology across sub-catchment	weighted count	Int32	−9999	USGS	30 arcsec	1960–2000
Surface geology	weighted sum	geology_weighted_sum.nc	72	geo_wsum_72	Quaternary geology across sub-catchment	weighted count	Int32	−9999	USGS	30 arcsec	1960–2000
Surface geology	weighted sum	geology_weighted_sum.nc	73	geo_wsum_73	Quaternary, Neogne geology across sub-catchment	weighted count	Int32	−9999	USGS	30 arcsec	1960–2000
Surface geology	weighted sum	geology_weighted_sum.nc	74	geo_wsum_74	Quaternary, Tertiary geology across sub-catchment	weighted count	Int32	−9999	USGS	30 arcsec	1960–2000
Surface geology	weighted sum	geology_weighted_sum.nc	75	geo_wsum_75	Salt geology across sub-catchment	weighted count	Int32	−9999	USGS	30 arcsec	1960–2000
Surface geology	weighted sum	geology_weighted_sum.nc	76	geo_wsum_76	Silurian geology across sub-catchment	weighted count	Int32	−9999	USGS	30 arcsec	1960–2000
Surface geology	weighted sum	geology_weighted_sum.nc	77	geo_wsum_77	Silurian, Cambrian geology across sub-catchment	weighted count	Int32	−9999	USGS	30 arcsec	1960–2000
Surface geology	weighted sum	geology_weighted_sum.nc	78	geo_wsum_78	Silurian, Ordovician geology across sub-catchment	weighted count	Int32	−9999	USGS	30 arcsec	1960–2000
Surface geology	weighted sum	geology_weighted_sum.nc	79	geo_wsum_79	Silurian, Proterozoic geology across sub-catchment	weighted count	Int32	−9999	USGS	30 arcsec	1960–2000
Surface geology	weighted sum	geology_weighted_sum.nc	80	geo_wsum_80	Tertiary geology across sub-catchment	weighted count	Int32	−9999	USGS	30 arcsec	1960–2000
Surface geology	weighted sum	geology_weighted_sum.nc	81	geo_wsum_81	Tertiary, Cretaceous geology across sub-catchment	weighted count	Int32	−9999	USGS	30 arcsec	1960–2000
Surface geology	weighted sum	geology_weighted_sum.nc	82	geo_wsum_82	Triassic geology across sub-catchment	weighted count	Int32	−9999	USGS	30 arcsec	1960–2000
Surface geology	weighted sum	geology_weighted_sum.nc	83	geo_wsum_83	Triassic, Carboniferous geology across sub-catchment	weighted count	Int32	−9999	USGS	30 arcsec	1960–2000
Surface geology	weighted sum	geology_weighted_sum.nc	84	geo_wsum_84	Triassic, Devonian geology across sub-catchment	weighted count	Int32	−9999	USGS	30 arcsec	1960–2000
Surface geology	weighted sum	geology_weighted_sum.nc	85	geo_wsum_85	Triassic, Mississippian geology across sub-catchment	weighted count	Int32	−9999	USGS	30 arcsec	1960–2000
Surface geology	weighted sum	geology_weighted_sum.nc	86	geo_wsum_86	Triassic, Ordovician geology across sub-catchment	weighted count	Int32	−9999	USGS	30 arcsec	1960–2000
Surface geology	weighted sum	geology_weighted_sum.nc	87	geo_wsum_87	Triassic, Paleozoic geology across sub-catchment	weighted count	Int32	−9999	USGS	30 arcsec	1960–2000
Surface geology	weighted sum	geology_weighted_sum.nc	88	geo_wsum_88	Triassic, Pennsylvanian geology across sub-catchment	weighted count	Int32	−9999	USGS	30 arcsec	1960–2000
Surface geology	weighted sum	geology_weighted_sum.nc	89	geo_wsum_89	Triassic, Permian geology across sub-catchment	weighted count	Int32	−9999	USGS	30 arcsec	1960–2000
Surface geology	weighted sum	geology_weighted_sum.nc	90	geo_wsum_90	Triassic, Proterozoic geology across sub-catchment	weighted count	Int32	−9999	USGS	30 arcsec	1960–2000
Surface geology	weighted sum	geology_weighted_sum.nc	91	geo_wsum_91	Unknown geology across sub-catchment	weighted count	Int32	−9999	USGS	30 arcsec	1960–2000
Surface geology	weighted sum	geology_weighted_sum.nc	92	geo_wsum_92	Water geology across sub-catchment	weighted count	Int32	−9999	USGS	30 arcsec	1960–2000
Soil	minimum	soil_minimum.nc	1	soil_min_01	Soil organic carbon (ORCDRC) across sub-catchment	[g/kg]	Int32	−999	ISRIC	30 arcsec	1950–2005
Soil	minimum	soil_minimum.nc	2	soil_min_02	Soil pH in H2O (PHIHOX) across sub-catchment	pH * 10	Int32	−999	ISRIC	30 arcsec	1950–2005
Soil	minimum	soil_minimum.nc	3	soil_min_03	Sand content mass fraction (SNDPPT) across sub-catchment	[%]	Int32	−999	ISRIC	30 arcsec	1950–2005
Soil	minimum	soil_minimum.nc	4	soil_min_04	Silt content mass fraction (SLTPPT) across sub-catchment	[%]	Int32	−999	ISRIC	30 arcsec	1950–2005
Soil	minimum	soil_minimum.nc	5	soil_min_05	Clay content mass fraction (CLYPPT) across sub-catchment	[%]	Int32	−999	ISRIC	30 arcsec	1950–2005
Soil	minimum	soil_minimum.nc	6	soil_min_06	Coarse fragments (>2 mm fraction) volumetric (CFRVOL) across sub-catchment	[%]	Int32	−999	ISRIC	30 arcsec	1950–2005
Soil	minimum	soil_minimum.nc	7	soil_min_07	Cation exchange capacity (CEC) across sub-catchment	[cmol/kg]	Int32	−999	ISRIC	30 arcsec	1950–2005
Soil	minimum	soil_minimum.nc	8	soil_min_08	Bulk density of the fine earth fraction (BLD) across sub-catchment	[kg/m3]	Int32	−999	ISRIC	30 arcsec	1950–2005
Soil	minimum	soil_minimum.nc	9	soil_min_09	Depth to bedrock (R horizon) up to maximum 240 cm (BDRICM) across sub-catchment	[cm]	Int32	−999	ISRIC	30 arcsec	1950–2005
Soil	minimum	soil_minimum.nc	10	soil_min_10	Predicted probability of occurence (0–100%) of R horizon (BDRLOG) across sub-catchment	[%]	Int32	−999	ISRIC	30 arcsec	1950–2005
Soil	maximum	soil_maximum.nc	1	soil_max_01	Soil organic carbon (ORCDRC) across sub-catchment	[g/kg]	Int32	−999	ISRIC	30 arcsec	1950–2005
Soil	maximum	soil_maximum.nc	2	soil_max_02	Soil pH in H2O (PHIHOX) across sub-catchment	pH * 10	Int32	−999	ISRIC	30 arcsec	1950–2005
Soil	maximum	soil_maximum.nc	3	soil_max_03	Sand content mass fraction (SNDPPT) across sub-catchment	[%]	Int32	−999	ISRIC	30 arcsec	1950–2005
Soil	maximum	soil_maximum.nc	4	soil_max_04	Silt content mass fraction (SLTPPT) across sub-catchment	[%]	Int32	−999	ISRIC	30 arcsec	1950–2005
Soil	maximum	soil_maximum.nc	5	soil_max_05	Clay content mass fraction (CLYPPT) across sub-catchment	[%]	Int32	−999	ISRIC	30 arcsec	1950–2005
Soil	maximum	soil_maximum.nc	6	soil_max_06	Coarse fragments (>2 mm fraction) volumetric (CFRVOL) across sub-catchment	[%]	Int32	−999	ISRIC	30 arcsec	1950–2005
Soil	maximum	soil_maximum.nc	7	soil_max_07	Cation exchange capacity (CEC) across sub-catchment	[cmol/kg]	Int32	−999	ISRIC	30 arcsec	1950–2005
Soil	maximum	soil_maximum.nc	8	soil_max_08	Bulk density of the fine earth fraction (BLD) across sub-catchment	[kg/m3]	Int32	−999	ISRIC	30 arcsec	1950–2005
Soil	maximum	soil_maximum.nc	9	soil_max_09	Depth to bedrock (R horizon) up to maximum 240 cm (BDRICM) across sub-catchment	[cm]	Int32	−999	ISRIC	30 arcsec	1950–2005
Soil	maximum	soil_maximum.nc	10	soil_max_10	Predicted probability of occurence (0–100%) of R horizon (BDRLOG) across sub-catchment	[%]	Int32	−999	ISRIC	30 arcsec	1950–2005
Soil	range	soil_range.nc	1	soil_range_01	Soil organic carbon (ORCDRC) across sub-catchment	[g/kg]	Int32	−999	ISRIC	30 arcsec	1950–2005
Soil	range	soil_range.nc	2	soil_range_02	Soil pH in H2O (PHIHOX) across sub-catchment	pH * 10	Int32	−999	ISRIC	30 arcsec	1950–2005
Soil	range	soil_range.nc	3	soil_range_03	Sand content mass fraction (SNDPPT) across sub-catchment	[%]	Int32	−999	ISRIC	30 arcsec	1950–2005
Soil	range	soil_range.nc	4	soil_range_04	Silt content mass fraction (SLTPPT) across sub-catchment	[%]	Int32	−999	ISRIC	30 arcsec	1950–2005
Soil	range	soil_range.nc	5	soil_range_05	Clay content mass fraction (CLYPPT) across sub-catchment	[%]	Int32	−999	ISRIC	30 arcsec	1950–2005
Soil	range	soil_range.nc	6	soil_range_06	Coarse fragments (>2 mm fraction) volumetric (CFRVOL) across sub-catchment	[%]	Int32	−999	ISRIC	30 arcsec	1950–2005
Soil	range	soil_range.nc	7	soil_range_07	Cation exchange capacity (CEC) across sub-catchment	[cmol/kg]	Int32	−999	ISRIC	30 arcsec	1950–2005
Soil	range	soil_range.nc	8	soil_range_08	Bulk density of the fine earth fraction (BLD) across sub-catchment	[kg/m3]	Int32	−999	ISRIC	30 arcsec	1950–2005
Soil	range	soil_range.nc	9	soil_range_09	Depth to bedrock (R horizon) up to maximum 240 cm (BDRICM) across sub-catchment	[cm]	Int32	−999	ISRIC	30 arcsec	1950–2005
Soil	range	soil_range.nc	10	soil_range_10	Predicted probability of occurence (0–100%) of R horizon (BDRLOG) across sub-catchment	[%]	Int32	−999	ISRIC	30 arcsec	1950–2005
Soil	average	soil_average.nc	1	soil_avg_01	Soil organic carbon (ORCDRC) across sub-catchment	[g/kg]	Int32	−999	ISRIC	30 arcsec	1950–2005
Soil	average	soil_average.nc	2	soil_avg_02	Soil pH in H2O (PHIHOX) across sub-catchment	pH * 10	Int32	−999	ISRIC	30 arcsec	1950–2005
Soil	average	soil_average.nc	3	soil_avg_03	Sand content mass fraction (SNDPPT) across sub-catchment	[%]	Int32	−999	ISRIC	30 arcsec	1950–2005
Soil	average	soil_average.nc	4	soil_avg_04	Silt content mass fraction (SLTPPT) across sub-catchment	[%]	Int32	−999	ISRIC	30 arcsec	1950–2005
Soil	average	soil_average.nc	5	soil_avg_05	Clay content mass fraction (CLYPPT) across sub-catchment	[%]	Int32	−999	ISRIC	30 arcsec	1950–2005
Soil	average	soil_average.nc	6	soil_avg_06	Coarse fragments (>2 mm fraction) volumetric (CFRVOL) across sub-catchment	[%]	Int32	−999	ISRIC	30 arcsec	1950–2005
Soil	average	soil_average.nc	7	soil_avg_07	Cation exchange capacity (CEC) across sub-catchment	[cmol/kg]	Int32	−999	ISRIC	30 arcsec	1950–2005
Soil	average	soil_average.nc	8	soil_avg_08	Bulk density of the fine earth fraction (BLD) across sub-catchment	[kg/m3]	Int32	−999	ISRIC	30 arcsec	1950–2005
Soil	average	soil_average.nc	9	soil_avg_09	Depth to bedrock (R horizon) up to maximum 240 cm (BDRICM) across sub-catchment	[cm]	Int32	−999	ISRIC	30 arcsec	1950–2005
Soil	average	soil_average.nc	10	soil_avg_10	Predicted probability of occurence (0–100%) of R horizon (BDRLOG) across sub-catchment	[%]	Int32	−999	ISRIC	30 arcsec	1950–2005
Soil	weighted average	soil_weighted average.nc	1	soil_wavg_01	Soil organic carbon (ORCDRC) across sub-catchment	[g/kg]	Int32	−999	ISRIC	30 arcsec	1950–2005
Soil	weighted average	soil_weighted average.nc	2	soil_wavg_02	Soil pH in H2O (PHIHOX) across sub-catchment	pH * 10	Int32	−999	ISRIC	30 arcsec	1950–2005
Soil	weighted average	soil_weighted average.nc	3	soil_wavg_03	Sand content mass fraction (SNDPPT) across sub-catchment	[%]	Int32	−999	ISRIC	30 arcsec	1950–2005
Soil	weighted average	soil_weighted average.nc	4	soil_wavg_04	Silt content mass fraction (SLTPPT) across sub-catchment	[%]	Int32	−999	ISRIC	30 arcsec	1950–2005
Soil	weighted average	soil_weighted average.nc	5	soil_wavg_05	Clay content mass fraction (CLYPPT) across sub-catchment	[%]	Int32	−999	ISRIC	30 arcsec	1950–2005
Soil	weighted average	soil_weighted average.nc	6	soil_wavg_06	Coarse fragments (>2 mm fraction) volumetric (CFRVOL) across sub-catchment	[%]	Int32	−999	ISRIC	30 arcsec	1950–2005
Soil	weighted average	soil_weighted average.nc	7	soil_wavg_07	Cation exchange capacity (CEC) across sub-catchment	[cmol/kg]	Int32	−999	ISRIC	30 arcsec	1950–2005
Soil	weighted average	soil_weighted average.nc	8	soil_wavg_08	Bulk density of the fine earth fraction (BLD) across sub-catchment	[kg/m3]	Int32	−999	ISRIC	30 arcsec	1950–2005
Soil	weighted average	soil_weighted average.nc	9	soil_wavg_09	Depth to bedrock (R horizon) up to maximum 240 cm (BDRICM) across sub-catchment	[cm]	Int32	−999	ISRIC	30 arcsec	1950–2005
Soil	weighted average	soil_weighted average.nc	10	soil_wavg_10	Predicted probability of occurence (0–100%) of R horizon (BDRLOG) across sub-catchment	[%]	Int32	−999	ISRIC	30 arcsec	1950–2005
Quality control	—	quality_control.nc	1	missing_cells	Cells that were filled based on the maximum neighbour value	—	Byte	−127	—	—	—
Quality control	—	quality_control.nc	2	cells_removed	Cells that were removed manually	—	Byte	−127	—	—	—
For each single variable we provide the variable category, type of upstream aggregation, band ID in the netCDF file, variable code, variable explanation, unit of measurement, datatype, NoData value, and the original source, native spatial grain and data acquisition period.											

**Table 3:** R^2^ values derived from the linear regression between observed monthly minimum and maximum stream temperature, and the upstream average and distance weighted averaged air temperature within the stream network, as well as between the observed monthly discharge and upstream sum and distance weighted sum of precipitation across the sub-catchments

**Month**	**Monthly minimum temperature**		**Monthly maximum temperature**		**Monthly discharge**	
	**Upstream average**	**Distance weighted average**	**Upstream average**	**Distance weighted average**	**Upstream sum**	**Distance weighted sum**
January	**0.57**	0.13	0.43	**0.44**	**0.86**	0.03
February	**0.57**	0.22	0.77	**0.78**	**0.87**	0.03
March	**0.72**	0.35	0.83	**0.84**	**0.89**	0.03
April	0.65	**0.71**	0.74	**0.75**	**0.84**	0.02
May	0.68	**0.74**	**0.66**	0.63	**0.71**	0.01
June	0.70	**0.75**	**0.59**	0.55	**0.52**	0.00
July	0.64	**0.69**	**0.58**	0.55	**0.30**	0.00
August	0.62	**0.68**	**0.62**	0.60	**0.23**	0.00
September	0.64	**0.71**	**0.76**	**0.76**	**0.42**	0.00
October	0.57	**0.64**	**0.73**	**0.73**	**0.72**	0.01
November	**0.59**	0.55	0.76	**0.77**	**0.83**	0.01
December	**0.61**	0.27	0.82	**0.84**	**0.85**	0.02
The monthly R^2^ values correspond to the single plots in the Figs 4 and 5, respectively. Higher values among each pair (average/sum versus weighted average/sum) are in bold.						
