# Supplementary material for: Near-global freshwater-specific environmental variables for biodiversity analyses in 1 km resolution
Source: Sci Data. 2015 Dec 8;2:150073. doi: 10.1038/sdata.2015.73 (PMC4672682; doi:10.1038/sdata.2015.73)
Supplement: Supplementary Information [file sdata201573-s1.docx]

***Near-global freshwater-specific environmental variables for biodiversity analyses in 1km resolution***

Sami Domisch^1^, Giuseppe Amatulli^1^, Walter Jetz^1^

^1^Department of Ecology and Evolutionary Biology, Yale University, 165 Prospect Street, New Haven, CT 06511, USA

Corresponding author: Sami Domisch ([sami.domisch@yale.edu](mailto:sami.domisch@yale.edu))

**Supplementary Information**

**Table of contents**

**Table 2** Full overview of all newly-developed freshwater-specific environmental variables (online only)

**Figure S1 a-b** Locations of all stations for the data validation ……………………………………………………………….. 2

**Figure S2** Observed and aggregated upstream temperatures ……………………………………………………………….. 3

**Example code** to load and process the netCDF files in R …….…………………………………………………………………. 4

**Example code** to generate stream-specific variables for a given study area in GRASS GIS ….…………………. 7

**Data citations** .………………………………………………………………………………………………………………………………………. 9

**References** ……………………………………………………………………………………………………………………………………………. 9

This dataset incorporates data from the HydroSHEDS database which is © World Wildlife Fund, Inc. (2006-2013). Portions of the HydroSHEDS database incorporate data which are the intellectual property rights of © USGS (2006-2008), NASA (2000-2005), ESRI (1992-1998), CIAT (2004-2006), UNEP-WCMC (1993), WWF (2004), Commonwealth of Australia (2007), and Her Royal Majesty and the British Crown. The HydroSHEDS database and more information are available at <http://www.hydrosheds.org>.

Portions of the Global Lakes and Wetlands Database (GLWD) incorporated in this dataset are the intellectual property rights of © Bernhard Lehner (World Wildlife Fund US, Center for Environmental Systems Research, 2004), Environmental

Systems Research Institute, Inc. (ESRI, 2004), and UNEP World Conservation Monitoring Centre (UNEP-WCMC, 2004). The GLWD database and more information are available at <http://www.worldwildlife.org/pages/global-lakes-and-wetlands-database>.

The citations for these datasets are:

Lehner, B., Verdin, K., & Jarvis, A. (2008). New global hydrography derived from spaceborne elevation data. EOS, Transactions American Geophysical Union, 89(10), 93-94.

*Lehner, B., & Döll, P. (2004). Development and validation of a global database of lakes, reservoirs and wetlands. Journal of Hydrology, 296(1), 1-22.*

**
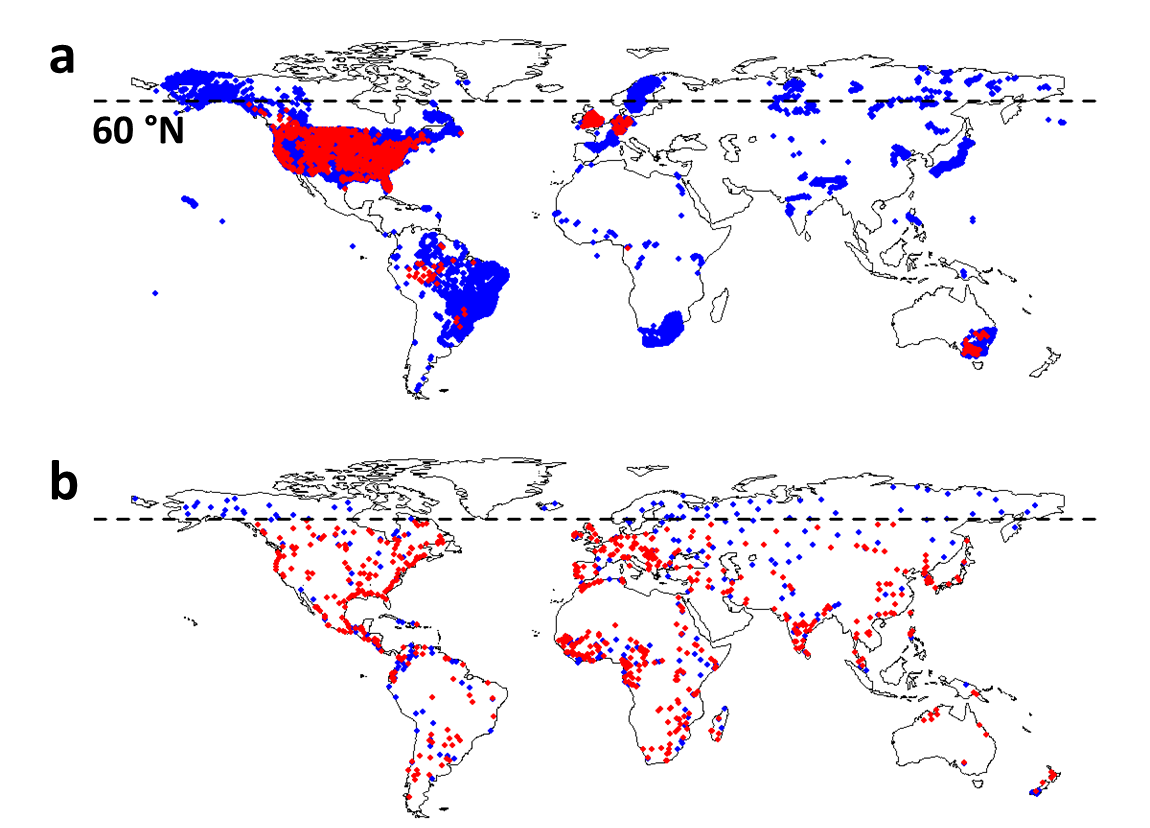
**

**Figure S1 a-b** Locations of all stations with (**a**) observed stream temperature (data from^1-3^) and (**b**) discharge (data from^4^). Blue points mark locations with any observed data, whereas red points represent those locations that were used for the validation by means of linear regression (Table 3, Figs. 4, 5). Only data below 60˚N latitude (dashed line) was used, as the HydroSHEDS hydrography^5^ does currently not exceed this area.


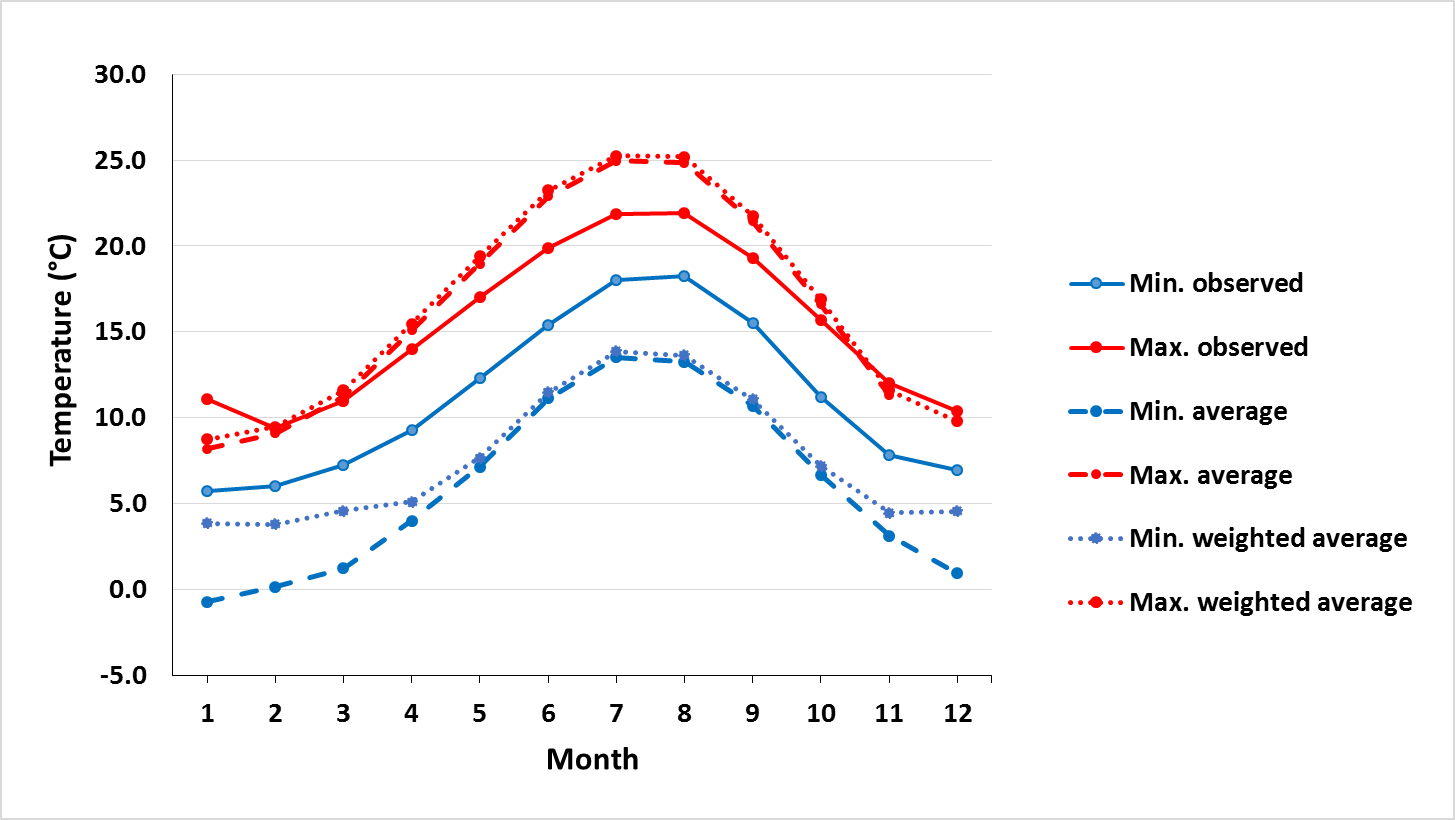


**Figure S2** Mean monthly minimum (blue) and maximum (red) temperature values derived from observed data (solid lines), upstream average (dashed lines) and weighted average (dotted lines) temperature variables (Data Citation 1). Observed stream temperature data derived from^1-3^, see Fig. S1A.

**Example code to load and process the variables in R**

Load, rename, crop and export the variables in R. For the netCDF-4 files the ncdf4 library is needed and depending on the operating system, it needs to be downloaded and installed "manually" (see below). See also other useful functions in the raster package, and additional code on <http://www.earthenv.org/streams> to snap points to the stream network, and extract the variables to the points.

**Install the packages and load libraries:**

install.packages("raster")
install.packages("ncdf4")

install.packages("rgdal")

install.packages("maps")

install.packages("foreach")

install.packages("doParallel")

### For Windows, download the "ncdf4" library and install locally.
### Here is an example for Windows 64-bit:
download.file("http://cirrus.ucsd.edu/~pierce/ncdf/win64/ncdf4_1.12.zip",
 paste(getwd(), "/ncdf4_1.12.zip", sep=""))
install.packages(paste(getwd(), "/ncdf4_1.12.zip", sep=""), repos=NULL)
library(raster); library(ncdf4); library(maps); library(foreach); library(doParallel)

**Example: Load all 12 land cover variables for "average percent upstream cover" into a raster brick, crop the brick to a smaller extent, write layers to disk and convert to a** data.frame **in parallel:**

### Download the average landcover variables from EarthEnv
download.file("http://data.earthenv.org/streams/landcover_average.nc",
 paste(getwd(), "landcover_average.nc", sep="/"), mode = "wb")
### Load the 12 layers into a raster brick

lc_avg <- brick("landcover_average.nc")
### Check the number of layers
nlayers(lc_avg)

### Check the metadata for units, scale factors etc.

**nc_open**("landcover_average.nc")

### Add layer names. See Table S1 or the ReadMe for the sequence of the single layers
names(lc_avg) <- paste(c("lc_avg"), sprintf("%02d", seq(1:12)), sep="_")
### Extract one layer, e.g. the "Evergreen Broadleaf Trees"
lc02 <- lc_avg[["lc_avg_02"]]

### Plot the world and draw extent for cropping below 60°N latitude:
x11(10.3); map('world'); abline(h=60, lty=5, lwd=2, col="red"); text(-176, 64, "60°N", col="red")
### Crop to smaller extent e.g. by clicking the upper left and lower right corners of the desired rectangle
(ext <- drawExtent())
### Alternatively set the extent by coordinates
# ext <- extent(c(5,8,30,35))

### Crop entire raster brick in parallel
### Make cluster object
cl <- makePSOCKcluster(detectCores()-2) # leave two cores for background processes

# cl <- makePSOCKcluster(1) # if old PC use only 1 core

registerDoParallel(cl) # register parallel backend
getDoParWorkers() # show number of workers

### Crop all layers in the brick and write the cropped layers to disk
lc_avg_crop <- foreach(i = iter(names(lc_avg)), .packages = c("raster", "ncdf4")) %dopar% {
 options(rasterNCDF4 = TRUE)
 tmp <- crop(lc_avg[[i]], ext, snap="in")
 filename=paste0(i, ".tif")
 writeRaster(tmp, filename=filename, overwrite=FALSE)
 }

### **foreach()** returns a list by default, get the layers back in a stack
lc_avg_crop <- stack(unlist(lc_avg_crop))
### Check the layers
plot(lc_avg_crop)

### Convert **raster** stack into a dataframe
lc_avg_crop_df <- foreach(i = iter(names(lc_avg_crop)), .combine=cbind.data.frame, .packages = c("raster")) %dopar% {
 as.data.frame(lc_avg_crop[[i]], na.rm=T)
 }

### Check output

head(lc_avg_crop_df)

summary(lc_avg_crop_df)

stopCluster(cl) # stop parallel backend

### Remove temporary raster-files on the hard disk
showTmpFiles()
removeTmpFiles()

**Load other variables:**

### Load elevation variables
elevation <- brick("elevation.nc")
### Add layer names
names(elevation) <- paste(c("dem"), c("min", "max", "range", "avg"), sep="_")

### Load slope variables
slope <- brick("slope.nc")
names(slope) <- paste(c("slope"), c("min", "max", "range", "avg"), sep="_")

### Load flow accumulation and stream length variables
flow_acc <- brick("flow_acc.nc")
names(flow_acc) <- paste(c("flow"), c("length", "acc"), sep="_")

### Load climate variables
tmin_avg <- brick("monthly_tmin_average.nc")
names(tmin_avg) <- paste(c("tmin_avg"), sprintf("%02d", seq(1:12)), sep="_")

tmax_avg <- brick("monthly_tmax_average.nc")
names(tmax_avg) <- paste(c("tmax_avg"), sprintf("%02d", seq(1:12)), sep="_")

prec_sum <- brick("monthly_prec_sum.nc")
names(prec_sum) <- paste(c("prec_sum"), sprintf("%02d", seq(1:12)), sep="_")

### Load long-term climate variables (temperature=average, precipitation=sum)
hydro_avg <- brick("hydroclim_average+sum.nc")
names(hydro_avg) <- paste(c("hydro_avg"), sprintf("%02d", seq(1:19)), sep="_")

### Load geological variables
geology <- brick("geology_weighted_sum.nc")
names(geology) <- paste(c("geo"), sprintf("%02d", seq(1:92)), sep="_")

### Load soil variables
soil_avg <- brick("soil_average.nc")
names(soil_avg) <- paste(c("soil_avg"), sprintf("%02d", seq(1:10)), sep="_")

**Example code to generate stream-specific variables for a given study area in GRASS GIS**

This example contains the following steps (see also the extended tutorial on [spatial-ecology.net](http://spatial-ecology.net/dokuwiki/doku.php?id=wiki:grassrivarvariable)):

- Download an exemplary digital elevation model (DEM)

- Run a hydrological conditioning of the DEM

- Extract the stream network from the DEM

- Calculate the sub-watersheds for each stream grid cell (*r.stream.watersheds*)

- Calculate contiguous stream-specific variables (*r.stream.variables*)

**Create and enter the folder where the data will be stored:**

!#/bin/bash
export INDIR=$HOME/grass_hydro
mkdir $INDIR
cd $INDIR

**Download and unzip a DEM from** [**WorldClim**](http://www.worldclim.org/tiles.php)**, and use it to create the GRASS GIS data base:**

wget -O $INDIR/alt_16_tif.zip "http://biogeo.ucdavis.edu/data/climate/worldclim/1_4/tiles/cur/alt_16_tif.zip"
unzip -o $INDIR/alt_16_tif.zip -d $INDIR
grass70 -text -c –e $INDIR/alt_16.tif $INDIR/grass_location

grass70 -text $INDIR/grass_location/PERMANENT # enter GRASS

**Import the DEM into GRASS:**

r.in.gdal input=$INDIR/alt_16.tif output=elevation

**Run hydrological conditioning:**

g.extension extension=r.hydrodem # install the r.hydrodem add-on
r.hydrodem input=elevation output=elevation_conditioned

**Download and install the *r.stream.watershed* and *r.stream.variables* add-ons:**

g.extension extension=r.stream.watersheds
g.extension extension=r.stream.variables

# Work-around in case the installation of the extensions causes problems. Download the add-ons and make them executable in the /addons –folder of GRASS (check the correct path on your system):

mkdir $HOME/.grass7/addons/scripts

# r.stream.watersheds:

wget -O $HOME/.grass7/addons/scripts/r.stream.watersheds "http://trac.osgeo.org/grass/export/66488/grass-addons/grass7/raster/r.stream.watersheds/r.stream.watersheds"

chmod 777 $HOME/.grass7/addons/scripts/r.stream.watersheds # make executable

# r.stream.variables:

wget -O $HOME/.grass7/addons/scripts/r.stream.variables "http://trac.osgeo.org/grass/export/66562/grass-addons/grass7/raster/r.stream.variables/r.stream.variables"

chmod 777 $HOME/.grass7/addons/scripts/r.stream.variables

### Other useful add-ons for hydrological applications:

### http://grasswiki.osgeo.org/wiki/Hydrological_Sciences

**Extract the stream network from the conditioned DEM. In this example, a minimum of 100 upstream cells are needed:**

r.watershed --h # see help regarding the options and flags

r.watershed elevation=elevation_conditioned drainage=drainage stream=stream threshold=100

**Add-on 1: Calculate the sub-watershed and sub-stream section for each stream grid cell using 4 processors:**

r.stream.watersheds stream=stream drainage=drainage cpu=4

**Add-on 2: Calculate stream-specific variables from the elevation layer:**

r.stream.variables variable=elevation output=cells,min,max,range,mean,stddev,coeff_var,sum area=watershed scale=1 cpu=4

### *Calculate the stream length (upstream cells within the river network):*r.stream.variables variable=elevation output=cells area=stream scale=1 cpu=4

**Export the stream network as a compressed GeoTIFF:**

r.out.gdal input=stream output=$INDIR/stream_network.tif type=Int32 nodata=-9999 createopt="COMPRESS=LZW,ZLEVEL=9"

### Data Citations

1. Domisch, S., Amatulli, G. & Jetz, W. *EarthEnv* <http://www.earthenv.org/streams> (2015)

**References**

1 Hartmann, J., Lauerwald, R. & Moosdorf, N. A Brief Overview of the GLObal RIver Chemistry Database, GLORICH. *Procedia Earth and Planetary Science* **10**, 23-27 (2014).

2 Environmental Agency: Surface Water Temperature Archive for England and Wales, available at <http://www.geostore.com/environment-agency/WebStore?xml=environment-agency/xml/ogcDataDownload.xml>.

3 National Water Quality Monitoring Council. Water quality data provided by USGS, EPA and USDA, available at <http://waterqualitydata.us/>.

4 Vorosmarty, C. J., Fekete, B. M. & Tucker, B. A. Global River Discharge, 1807-1991, V. 1.1 (RivDIS). Data set. Available online [<http://www.daac.ornl.gov>] from Oak Ridge National Laboratory Distributed Active Archive Center, Oak Ridge, TN, U.S.A. (1998).

5 Lehner, B., Verdin, K. & Jarvis, A. New global hydrography derived from spaceborne elevation data. *Eos, Transactions, AGU* **89**, 93–94 (2008).
